# Combination of bacteriophage–probiotics alleviates intestinal barrier dysfunction by regulating gut microbiome in a chick model of multidrug-resistant *Salmonella* infection

**DOI:** 10.1186/s40104-025-01324-4

**Published:** 2026-01-23

**Authors:** Youbin Choi, Anna Kang, Eunsol Seo, Daniel Junpyo Lee, Junha Park, Yeonsoo Kim, Keesun Yu, Cheol‑Heui Yun, Ki Beom Jang, Woo Kyun Kim, Kwanseob Shim, Darae Kang, Younghoon Kim

**Affiliations:** 1 Department of Agricultural Biotechnology and Research Institute of Agriculture and Life Science, Seoul National University, Seoul, 08826 Korea; 2 Department of Poultry Science, University of Georgia, Athens, GA 30602 USA; 3 Department of Animal Biotechnology, Jeonbuk National University, Jeonju, 54896 Korea

**Keywords:** Antibiotic alternatives, Gut microbiome, Intestinal barrier, Metabolites, Multidrug-resistant *Salmonella*, Phage–probiotic combination

## Abstract

**Background:**

The rapid emergence of multidrug-resistant *Salmonella* in poultry demands alternative control strategies beyond conventional antibiotics. In this study, we evaluated a combination of lytic *Salmonella*-infecting bacteriophages (SLAM_phiST45 and SLAM_phiST56) and a probiotic bacterium *Limosilactobacillus reuteri* (SLAM_LAR11) in a chick model challenged with *Salmonella enterica* serovar Typhimurium infection.

**Results:**

Co-administration with two-phage cocktail and a probiotic showed markedly reduced *Salmonella* colonization in the gut and systemic organs of chicks, comparable to the effect of phage-only treatment. In contrast with phage-only treatment, the combined therapy significantly improved the rate of body-weight change from the day of infection to necropsy (*P* < 0.0001) and alleviated infection-associated splenomegaly (*P* = 0.028) and hepatomegaly (*P* = 0.011). In the ileum, the villus height-to-crypt depth ratio (VH/CD) increased significantly (*P* = 0.044). In the colon, expression of tight-junction genes *OCLN* (*P* = 0.014), *TJP1* (*P* < 0.0001), and *MUC2* (*P* = 0.011) was elevated, whereas the pro-inflammatory cytokine *IL6* was reduced (*P* = 0.018). These improvements were accompanied, in the cecum, by trends toward decreases in *Escherichia–Shigella* (*P* = 0.09) and *Clostridium* (*P* = 0.16) and a trend toward an increase in *Blautia* (*P* = 0.11); additionally, in the ileum, *Lactobacillus* (*P* = 0.037) and *Blautia* (*P* = 0.016) increased significantly, yielding a more balanced microbiota than with phage-only treatment. Consistently, levels of functional metabolites, including acetic acid (LDA = 3.32) and lactic acid (LDA = 5.29), were increased.

**Conclusion:**

Taken together, these findings demonstrate that phage–probiotic co-administration not only enhances the clearance of multidrug-resistant *Salmonella* more effectively than phage treatment alone but also promotes intestinal health, highlighting its potential as an antibiotic-alternatives strategy to improve intestinal health and ensure food safety in poultry production systems.

**Graphical Abstract:**

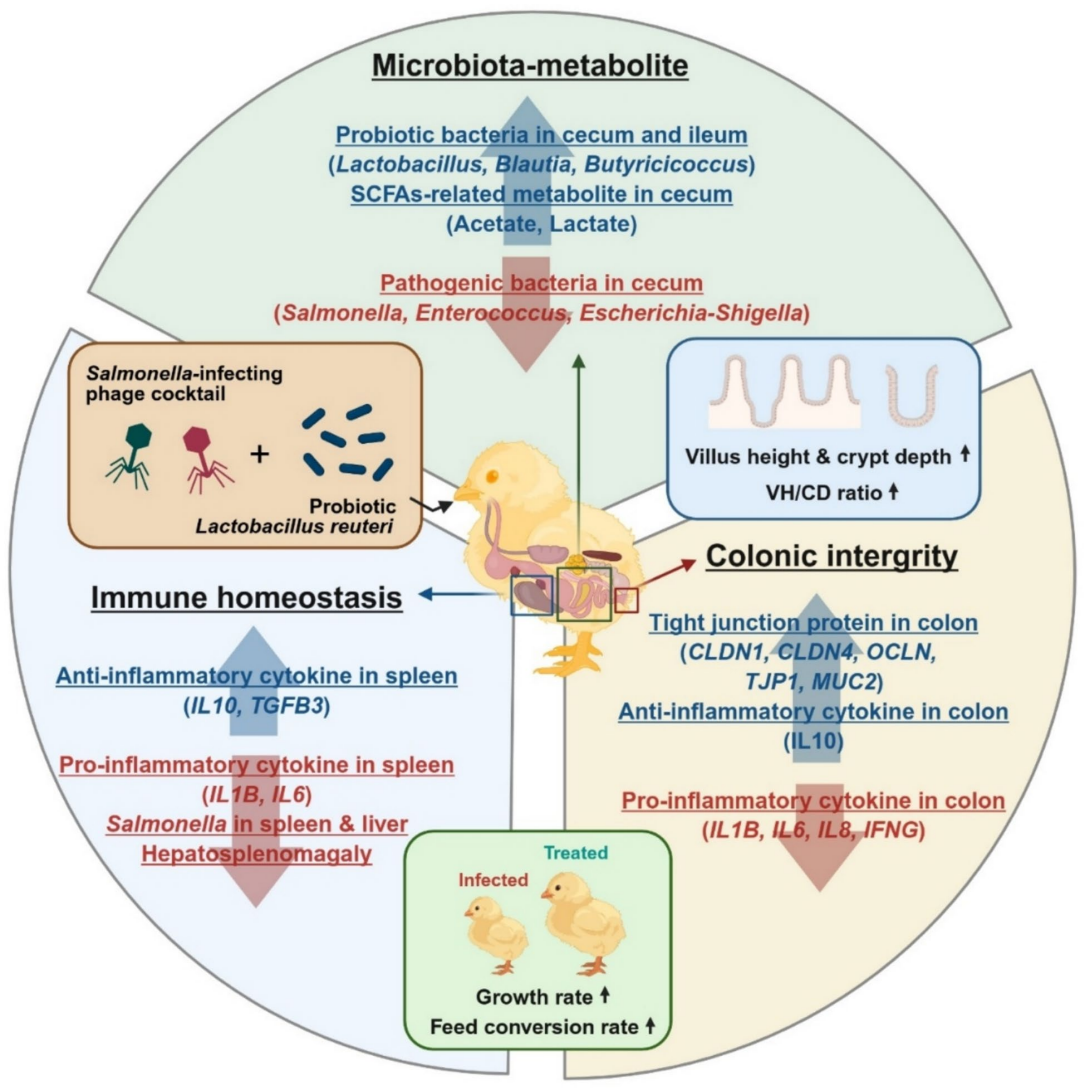

**Supplementary Information:**

The online version contains supplementary material available at 10.1186/s40104-025-01324-4.

## Introduction

*Salmonella* continues to be one of the leading causes of foodborne illness globally, with poultry products serving as a major transmission route to humans [1]. Among non-typhoidal serovars, *Salmonella enterica* serovar Typhimurium commonly colonize the intestinal tract of chickens [2]. The infection has been shown to compromise intestinal barrier function with increased inflammatory response as well as impaired growth performance in poultry [3, 4]. Birds infected with *Salmonella* frequently exhibit hepatosplenomegaly, elevated expression of pro-inflammatory cytokines, and reduced expression of tight junction and mucin genes [5]. Moreover, widespread antibiotic use in the poultry industry has contributed to the emergence and dissemination of multidrug-resistant (MDR) *Salmonella* strains [6]. In response to growing public health and safety concerns, many countries have banned the use of antibiotic growth promoters in livestock production systems [7]. Therefore, there is increasing interest in developing alternative strategies to control *Salmonella* colonization in poultry.

Bacteriophages (phages) are viruses that selectively infect and lyse specific bacteria, enabling targeted elimination of pathogenic species without broadly disrupting the gut microbiota. Phage therapy has received considerable attention due to their self-replicating characteristics and narrow host range, which could be an effective biocontrol method [8]. Several studies have demonstrated that phage administration could effectively reduce populations of pathogenic *Escherichia coli*, *Salmonella*, and *Clostridium*, while promoting populations of beneficial microbes in poultry [9–11]. However, despite its potential, phage therapy faces challenges in maintaining durable efficacy, primarily due to the rapid emergence of phage-resistant bacterial populations. In Gram-negative bacteria, such resistance is commonly driven by modifications or loss of surface receptors such as capsules, lipopolysaccharides (LPS), outer membrane proteins, and flagella that are essential for phage attachment [12]. For flagellotropic (Chi-like) phages that depend on flagellar expression and rotation, phase variation or reduced motility can readily confer resistance [13].

Consequently, phage cocktails targeting multiple, non-overlapping receptors or generalist phages with multi-receptor binding have been proposed to delay resistance, although phage–phage interactions (e.g., synergistic or antagonistic effects) and the optimal composition/dosing remain unsettled [14, 15]. In parallel, combination regimens involving antibiotics or adjuvants have also shown promise in enhancing phage efficacy and mitigating resistance [16]. While many phages have narrow host ranges, perturbations in the target population can induce community-level and metabolomic shifts, as demonstrated in model systems such as defined consortia in chemostats and gnotobiotic mice [16]. In contrast, clinical observations have reported minimal microbiota disruption under some contexts [17]; hence, these observations emphasize the importance of integrating microbiome and metabolome assessments into future phage-based applications to ensure efficacy while minimizing ecological disruptions [18].

Probiotics represent a complementary strategy to promote intestinal health by modulating microbial communities and enhancing host immune responses [19]. In poultry production systems, probiotic supplementation has been associated with improved gut barrier function, enhanced growth performance, and decreased *Salmonella* colonization or shedding [20, 21]. Lactic acid bacteria such as *Lactobacillus*, *Bifidobacterium*, and *Enterococcus* can limit *Salmonella* through complementary mechanisms including competitive exclusion, production of antimicrobial components (e.g., organic acids and bacteriocins), and immune modulation [22–24]. Given these complementary modes of action, the combination of phages with probiotics is a rational strategy to mitigate infection-associated risks: phages rapidly debulk the target pathogen, while probiotics help stabilize the community and hinder recolonization [25]. In poultry production models, co-supplementation of a phage cocktail and a commercial probiotic improved growth performance and enriched short-chain fatty acid (SCFA)-associated taxa compared with either treatment alone [26]. Similarly, in a murine model of *Salmonella*-induced colitis, a phage cocktail combined with *Limosilactobacillus reuteri* outperformed either treatment alone by reducing pathogen burden, improving intestinal barrier integrity, and increasing fecal SCFA concentrations [27]. Consistent with these observations, evidence in broiler chickens shows that *L. reuteri* enhances mucosal immunity, attenuates pro-inflammatory cytokines, and reinforces epithelial barrier function via tight-junction gene induction [28, 29].

Based on previous findings, we therefore hypothesize that prophylactic (pre-challenge) co-administration of bacteriophages and probiotics provides superior protection against pathogenic bacterial infection compared to either treatment alone, by reducing the pathogen burden and maintaining intestinal microbiota balance. To test this hypothesis, we evaluate the in vivo efficacy of prophylactic phage–probiotic co-administration in a chick model challenged with multidrug-resistant *S*. Typhimurium.

## Materials and methods

### Bacteriophages, probiotic strain and pathogen

Bacteriophages SLAM_phiST45 and SLAM_phiST56 and the probiotic bacterium *Limosilactobacillus reuteri* SLAM_LAR11 (hereafter LAR11) were used in this study. These components were previously validated as an effective cocktail against *Salmonella* in the preliminary in vitro study [30]. *Salmonella enterica* serovar Typhimurium KVCC-BA0000422 (hereafter ST422), the pathogenic strain used for challenge, was originally isolated from a diseased chicken and obtained from the Korea Veterinary Culture Collection (KVCC, Gimcheon, Korea). The probiotic strain LAR11 was isolated from a broiler intestinal sample using a multi-omics approach and was selected based on a comprehensive evaluation of probiotic characteristics. Bacteriophages SLAM_phiST45 and SLAM_phiST56 were selected for their broad host range and synergistic anti-*Salmonella* activity in liquid culture, as demonstrated in previous work. All strains have been deposited in NCBI GenBank (phage accession numbers PP948674.1 and PP948675.1; the accession number for LAR11 is CP196337).

### Whole-genome sequencing and analysis of ST422 and LAR11

Whole-genome sequencing (WGS) was performed to characterize and confirm the identities of ST422 and LAR11. For each strain, a single colony was suspended in 1 mL of sterile water and genomic DNA (gDNA) extracted using the HiGene™ Genomic DNA Prep Kit (BioFACT, Korea). DNA libraries were prepared following the Oxford Nanopore Technologies (ONT) ligation sequencing kit protocol (SQK-LSK109) and sequenced on a MinION Mk1B (R9.4.1 flow cell) [31]. Reads longer than 1,000 bp were assembled de novo into a single contig using Flye v2.9.5 [32]. Gene annotation was conducted with Prokka v1.14.6 [33]. Functional gene analysis (including sequence alignments) was performed using ClustalW (default settings) in MEGA X v10.2.6 [34]. Nucleotide sequence identity was confirmed using NCBI BLASTn. Circular genome maps were generated using the CGView Server (Proksee.ca) [35].

### Animal administrations

All animal procedures followed institutional guidelines and were approved by the Jeonbuk National University (JBNU) IACUC (Approval No. JBNU 2022-091). A total of 100 one-day-old unsexed broiler chicks were obtained from a commercial hatchery (Harim, Korea). The supplier operates under HACCP certification (Harim PS Hatchery; certificate No. 2012-0-0446, valid through Oct 3, 2027) and maintains food-safety systems including HACCP/FSSC 22000. Upon arrival, the chicks were randomly divided into 5 groups (20 birds/group) and housed under standard rearing conditions, with each group placed in 5 pens containing 4 birds per pen. The brooding temperature was gradually reduced from 34 °C on d 1 to 32 °C by d 7, and then further reduced from 32 °C on d 8 to 28 °C by the end of the experiment. All birds had ad libitum access to feed and water. A commercial starter diet (Happy Chick Starter, Harim, Korea; crumble form) was provided from d 1 to d 12. According to the manufacturer’s specification (as-fed basis), the diet contained crude protein, 19.5%; crude fat, 3.0%; crude fiber, 5.5%; ash, 8.0%; calcium, 0.8%; metabolizable energy, 2,920 kcal/kg; methionine + cystine, 0.8%; and phosphorus, 0.9%. Primary ingredients included grains, oilseed meals, cereal by-products, meat and bone meal, limestone, dicalcium phosphate, refined salt, added amino acids (DL-methionine, L-lysine HCl, L-threonine), trace minerals (e.g., copper, zinc), enzyme additives (xylanase, phytase), vitamins (e.g., vitamins A and E), choline chloride, propionic acid, and animal fat/molasses.

### Experimental design

The experimental groups were classified according to treatment as follows. The BL group (uninfected control) received 1 mL of SM buffer (50 mmol/L Tris-HCl, 100 mmol/L NaCl, 10 mmol/L MgSO_4_, pH 7.5) and was not challenged with *Salmonella*. The SA group (infected control) received SM buffer and was orally challenged with 1 mL of ST422 (~ 1 × 10^9^ CFU/mL). The PR group (probiotic only) was administered 1 mL of LAR11 culture (~ 1 × 10^9^ CFU/mL) once daily for 3 d prior to challenge. The PC group (phage only) received a 1-mL phage cocktail composed of SLAM_phiST45 and SLAM_phiST56 (each ~ 1 × 10^9^ PFU/mL) prior to challenge. The PP group (combined treatment) was administered LAR11 together with the phage cocktail before challenge. For the combination preparation, 1 mL of LAR11 culture was centrifuged (3,220 × *g*, 15 min), and the resulting cell pellet was resuspended in 1 mL of the phage cocktail (500 µL each of SLAM_phiST45 and SLAM_phiST56). On d 7, all birds—except those in the BL group—were orally challenged with *Salmonella*. Five days post-challenge (d 12), all birds were humanely euthanized for sample collection. Body weights of the chicks were measured every other day starting from d 1, with the d 11 measurement replaced by d 12. Weight change was calculated between d 7 (pre-challenge) and d 12 (5 d post-challenge). Percent weight gain was calculated for each bird using the following formula:

$$\text{Weight gain},\%=\left(\left[{\mathrm{Weight}}_\mathrm{final}-{\mathrm{Weight}}_\mathrm{initial}\right]/{\mathrm{Weight}}_\mathrm{initial}\right)\times100$$

**Fig. 1:**
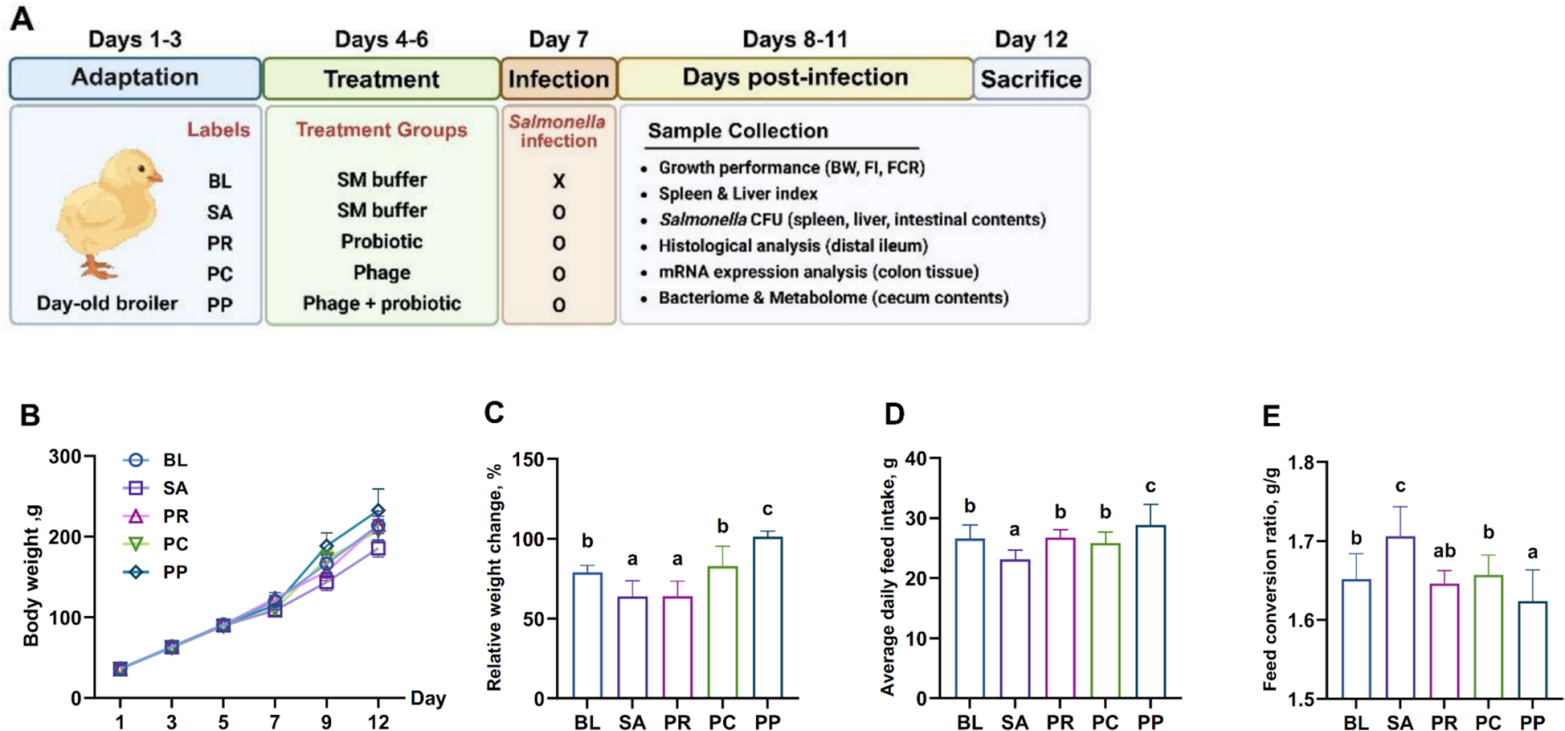
Experimental design and growth performance. **A** Schematic overview. One hundred day-old broiler chicks were randomly allocated into 5 groups (*n* = 20 per group), with each group further distributed into 5 pens (4 birds per pen). After a 3-day adaptation period (d 1–3), birds received respective treatments from d 4 to 6, followed by oral *Salmonella* challenge on d 7. All birds were euthanized on d 12 for subsequent analyses. **B** Body weight. Body weight was measured every other day starting on d 1 post-hatch. **C** Percent body weight gain from d 7 to d 12. *Salmonella* infection significantly stunted growth (SA), whereas phage (PC) and especially phage–probiotic (PP) treatments improved weight gain (PP showed the greatest gain). **D** Average daily feed intake (ADFI). For each group (*n* = 5 pens; 4 birds per pen), ADFI was calculated at the pen level as the total feed consumed over the 12-day experimental period divided by the number of days (12) and the number of birds in the pen, and expressed as g/bird/d. Group values represent the mean of pen-level ADFI; individual pens constitute biological replicates (*n* = 5 per group). **E** Feed conversion ratio (FCR). FCR was computed at the pen level as ADFI divided by average daily gain (ADG) of the same pen over the 12-day period. ADG was derived as [(body weight on d 12 − body weight on d 1)/12] and expressed as g/bird/d. Group values represent the mean of pen-level FCR; individual pens constitute biological replicates (*n* = 5 per group)

From each group, one chick was randomly selected from each of the 5 pens (total of 5 replicates per group) for microbiological, histological, and molecular analyses. A schematic overview of the experimental design is presented in Fig. 1A.

### Bacterial load of *Salmonella* in liver and spleen

For each bird, the liver index and spleen index were calculated as the organ weight relative to body weight. To determine *Salmonella* colonization of these organs, approximately 0.1 g of liver and 0.1 g of spleen from each selected bird were homogenized in 1 mL of phosphate-buffered saline (PBS) buffer (LPS solution, Korea) using bead-beating. The homogenates were filtered through a 40-µm cell strainer to remove debris. The filtrates were serially diluted in PBS buffer and plated on Xylose Lysine Deoxycholate (XLD) agar (BD Difco, USA) selective for *Salmonella*. Plates were incubated at 37 °C overnight, and *Salmonella* colonies were counted to determine colony-forming units (CFU) per gram of tissue.

### Splenic cytokine expression analysis

Spleen tissues from euthanized birds in the PC and PP groups were collected to compare pro- and anti-inflammatory cytokine expression levels. Total RNA was extracted using TRIzol™ reagent (Thermo Fisher Scientific, USA), and complementary DNA (cDNA) was synthesized using the iScript™ cDNA Synthesis Kit (Bio-Rad, USA). qRT-PCR was performed with the RealHelix™ [Green] qPCR Kit (Nanohelix, Korea). *GAPDH* was used as the internal control gene, and relative expression levels were calculated using the 2^–ΔΔCT^ method. The specific primer sequences [36] used for qRT-PCR are as follows: *GAPDH* (forward: 5′-TGCTGCCCAGAACATCATCC-3′, reverse: 5′-ACGGCAGGTCAGGTCAACAA-3′); *IL1B* (forward: 5′-ATGACCAAACTGCTGCGGAG-3′, reverse: 5′-AGGTGACGGGCTCAAAAACC-3′); *IL6* (forward: 5′-GACGAGGAGAAATGCCTGACG-3′, reverse: 5′-CCGAGTCTGGGATGACCACTTC-3′); *IL10* (forward: 5′-TCTACACAGATGAGGTCCTGCC-3′, reverse: 5′-AGGTGAAGAAGCGGTGACAG-3′); *TGFB3* (forward: 5′-CTCTGGGAGTTGCTTTGACGAC-3′, reverse: 5′-ACTGCTCTTTCTCATTCCTTGCC-3′).

### Quantification of *Salmonella* load in intestinal contents

Approximately 500 mg of content from the cecum, ileum, and jejunum of each selected bird was collected at necropsy and homogenized (with bead-beating). Total gDNA was extracted from each sample using the DNeasy PowerSoil Pro Kit (Qiagen, Germany). The relative abundance of *Salmonella* in the intestinal microbiota was quantified by SYBR Green-based quantitative PCR (qPCR) targeting the *Salmonella invA* gene (target) and the universal 16S rRNA gene (reference for total bacteria). The 2^–ΔΔCT^ method was used to calculate relative *Salmonella* levels. Primer sequences were: 16S rRNA gene (forward: 5′-CGGCAACGAGCGCAACCC-3′, reverse: 5′-CCATTGTAGCACGTGTGTAGCC-3′) and *invA* (forward: 5′-GCTGCTTTCTCTACTTAAC-3′, reverse: 5′-GTAATGGAATGACGAACAT-3′) [37].

### Histological analysis of the distal ileum

Distal ileum samples were collected and rinsed with sterile PBS buffer. Tissues were fixed in 4% neutral-buffered formalin, then dehydrated and embedded in paraffin. Sections were cut and stained with hematoxylin and eosin (H&E) using standard histological techniques [38]. Villus height and crypt depth were measured under light microscopy, and the villus height-to-crypt depth ratio was calculated for each sample. For each bird, multiple villi and crypts were measured from representative sections and averaged to yield a single value per individual.

### Colonic barrier integrity and inflammatory markers

Colonic tissue (colorectum) was sampled from the segment extending from the cecal junction (just distal to the paired ceca) to the cloaca, explicitly excluding cecal tissue. Total RNA was extracted using TRIzol™ reagent (Thermo Fisher Scientific, USA), and first-strand cDNA was synthesized with the iScript™ cDNA Synthesis Kit (Bio-Rad, USA). qRT-PCR was performed using the RealHelix™ [Green] qPCR Kit (Nanohelix, Korea) to measure mRNA expression of tight junction proteins and cytokines. *GAPDH* was used as the internal control gene, and relative expression was calculated by the 2^–ΔΔCT^ method. The primer sequences [36, 39] used for qRT-PCR analysis were as follows: *GAPDH* (forward: 5′-TGCTGCCCAGAACATCATCC-3′, reverse: 5′-ACGGCAGGTCAGGTCAACAA-3′); *CLDN1* (forward: 5′-TGGAGGATGACCAGGTGAAGA-3′, reverse: 5′-CGAGCCACTCTGTTGCCATA-3′); *CLDN4* (forward: 5′-GAAGCGCTGAACCGATACCA-3′, reverse: 5′-TGCTTCTGTGCCTCAGTTTCC-3′); *OCLN* (forward: 5′-ACGGCAGCACCTACCTCAA-3′, reverse: 5′-GGGCGAAGAAGCAGATGAG-3′); *TJP1* (forward: 5′-TATGAAGATCGTGCGCCTCC-3′, reverse: 5′-GAGGTCTGCCATCGTAGCTC-3′); *MUC2* (forward: 5′-TTCATGATGCCTGCTCTTGTG-3′, reverse: 5′-CCTGAGCCTTGGTACATTCTTGT-3′); *IL1B* (forward: 5′-ATGACCAAACTGCTGCGGAG-3′, reverse: 5′-AGGTGACGGGCTCAAAAACC-3′); *IL6* (forward: 5′-GACGAGGAGAAATGCCTGACG-3′, reverse: 5′-CCGAGTCTGGGATGACCACTTC-3′); *IL8* (forward: 5′-CTCTGTCGCAAGGTAAGTGAATCC-3′, reverse: 5′-CACACATCTCAGCAAGTGCCAAG-3′); *IL10* (forward: 5′-TCTACACAGATGAGGTCCTGCC-3′, reverse: 5′-AGGTGAAGAAGCGGTGACAG-3′); and *IFNG* (forward: 5′-AAGTCAAAGCCGCACATCAAAC-3′, reverse: 5′-CTGGATTCTCAAGTCGTTCATCG-3′).

### Microbiome analysis

Intestinal content samples were collected from the cecum, ileum, and jejunum for 16S rRNA gene sequencing. Cecal samples from all 5 groups (BL, SA, PR, PC, PP) were analyzed, while ileal and jejunal samples were analyzed only for the PC and PP groups using the same procedures. Approximately 500 mg of each sample was homogenized (with bead-beating), and gDNA was extracted using the DNeasy PowerSoil Pro Kit (Qiagen, Germany). The V3–V4 hypervariable region of the 16S rRNA gene was PCR-amplified using primers 515 F (5′-TCGTCGGCAGCGTCAGATGTGTATAAGAGACAGGTGCCAGCMGCCGCGGTAA-3′) and 806R (5′-GTCTCGTGGGCTCGGAGATGTGTATAAGAGACAGGGACTACHVGGGTWTCTAAT-3′), which include Illumina adapter overhang sequences. Amplicons were sequenced (paired-end 2 × 300 bp) on an Illumina NextSeq platform by Sanigen company (Anyang, Korea) [40–42].

### Metabolome analysis

Untargeted metabolomic analysis of cecal contents was performed as described previously [43]. Briefly, cecal samples were mixed with cold methanol, vortexed, and centrifuged at 13,420 × *g* for 10 min at 4 °C. The supernatant was filtered (0.22 µm) and dried in a vacuum concentrator. For derivatization, dried extracts were first incubated with pyridine containing 20 mg/mL methoxyamine hydrochloride (Sigma-Aldrich, USA) at 30 °C for 90 min, then treated with N,O-bis(trimethylsilyl)trifluoroacetamide (Sigma-Aldrich, USA) at 60 °C for 30 min. Metabolite profiling was conducted by gas chromatography–mass spectrometry (GC–MS) using a Thermo Trace 1310 gas chromatograph coupled to a Thermo ISQ LT single-quadrupole mass spectrometer (Thermo Fisher Scientific, USA). Data acquisition and peak quantification were performed using Thermo Xcalibur software. Metabolites were identified by matching spectra and retention indices to the NIST 2.0 library. The resulting data were normalized and analyzed using MetaboAnalyst 6.0 [44]. Prior to statistical analysis, data were log-transformed and auto-scaled to reduce heteroscedasticity. Multivariate analyses, including hierarchical clustering heatmaps, partial least squares discriminant analysis (PLS-DA), and sparse PLS-DA, were conducted to compare metabolic profiles between groups. Model reliability for PLS-DA was evaluated through tenfold cross-validation and 2,000-time permutation testing to prevent overfitting. Differential metabolite features between the PC and PP groups were identified using LEfSe, considering a linear discriminant analysis (LDA) score > 2.0 as significant [45].

### Correlation analysis between microbiota and metabolite profile in cecal contents

All statistical analyses were performed in R (version 4.x) [46]. Microbial relative abundance data (16S rRNA gene sequencing outputs) were treated as compositional and thus underwent a centered log-ratio (CLR) transformation to mitigate bias from differing library sizes. Metabolite concentration data were log_10_-transformed to approximate normality and reduce heteroscedasticity. We then computed Spearman’s rank correlation coefficients between the CLR-transformed abundances of selected bacterial genera and the log-transformed levels of each metabolite, as Spearman correlation is non-parametric and robust to non-linear relationships. The resulting correlation matrices (grouped by treatment) were visualized as heatmaps using the ComplexHeatmap package in R [47]. Each heatmap displayed correlation coefficients with a diverging color scale (e.g., blue for negative, red for positive correlations), and rows/columns were organized by hierarchical clustering or predefined order of biological interest. This analytical approach allowed us to identify and compare distinct microbiota–metabolite association patterns across the experimental groups in a clear, interpretable format.

### Statistical analysis

Unless otherwise noted, all experiments were performed with 5 biological replicates per treatment group, with one bird randomly selected from each of the 5 pens per group. Statistical analyses were conducted using GraphPad Prism (v10.3.0) [48]. In general, two-way analysis of variance (ANOVA) was used to evaluate differences among groups, followed by Tukey’s multiple comparison test. For microbiota (16S rRNA) data, α-diversity indices and the relative abundances of *Lactobacillus* and *Enterococcus* across all 5 groups were analyzed using two-way ANOVA, followed by Tukey’s multiple comparison test. Comparisons limited to the PC and PP groups, including *Escherichia–Shigella* and *Clostridium*, were evaluated using unpaired two-tailed *t*-tests. In all analyses, *P* < 0.05 was regarded as statistically significant, and data are presented as mean ± standard error of the mean (SEM).

## Results

### Genomic characterization of probiotic and pathogenic strains

Prior to conducting the in vivo experiments, the two phages included in the therapeutic cocktail were subjected to WGS and bioinformatic analysis [30]. Both phages were confirmed to be virulent phages that lack genes associated with horizontal gene transfer, antibiotic resistance, or known virulence factors (e.g., integrases or toxin genes), indicating their genomic safety for further experiments.

The *S.* Typhimurium strain ST422, used for challenge infection, was characterized as a multidrug-resistant (MDR) pathogen. Comprehensive Antibiotic Resistance Database (CARD) analysis identified a total of 45 antibiotic resistance genes (ARGs) in its genome, spanning eight antibiotic classes. These included resistance determinants against fosfomycin, macrolides, tetracyclines, aminoglycosides, β-lactams, polymyxins, glycopeptides, and bacitracin, as well as several genes encoding multidrug efflux pumps (Fig. S1A and Table S1).

In contrast, the genome of the probiotic *L. reuteri* strain LAR11 encoded multiple genetic features associated with probiotic function, including stress tolerance, gut colonization, biosynthesis of beneficial metabolites, and antimicrobial compound production. Notably, CARD analysis detected the presence of the *vanT* gene in LAR11, which confers an intrinsic resistance to glycopeptide antibiotics such as vancomycin. According to the European Food Safety Authority (EFSA) guidelines [49], such vancomycin resistance in certain lactobacilli is considered intrinsic, chromosomally encoded, and non-transferable, and therefore does not raise safety concerns for probiotic use (Fig. S1B).

### Phage–probiotic treatment enhances growth performance

During the first week, no significant differences were observed among groups; however, after *Salmonella* infection on d 7, the percent weight gain over the 5 d before necropsy differed significantly among treatments (Fig. 1B). Compared with the BL group, both the SA (*P* = 0.0015) and PR groups (*P* = 0.0017) showed a significant reduction in percent weight gain. In contrast, the PP group exhibited the highest weight gain among all treatments, showing a significant increase compared with the BL group (*P* < 0.0001). The PC group did not differ significantly from BL (*P* = 0.80), but showed markedly higher gains than the SA (*P* < 0.0001) and PR groups (*P* < 0.0001) (Fig. 1C). From d 1 to 12, average daily feed intake (ADFI) did not differ significantly among BL, PR, and PC groups; however, all three groups showed significantly higher ADFI compared with the SA group (*P* = 0.0003, *P* = 0.0002, *P* = 0.0029, respectively) and significantly lower ADFI than the PP group (*P* = 0.012, *P* = 0.019, *P* = 0.0011, respectively) (Fig. 1D). Feed conversion ratio (FCR) did not differ significantly among BL, PR, and PC groups, but all three showed significantly lower FCR values compared with the SA group (*P* = 0.0002, *P* < 0.0001, *P* = 0.0005, respectively). The PP group exhibited the highest FCR among all treatments, showing a significant increase compared with SA (*P* < 0.0001), BL (*P* = 0.05), and PC (*P* = 0.017), while no significant difference was observed compared with the PR group (*P* = 0.16) (Fig. 1E).

### Phage–probiotic treatment enhances immune homeostasis

**Fig. 2:**
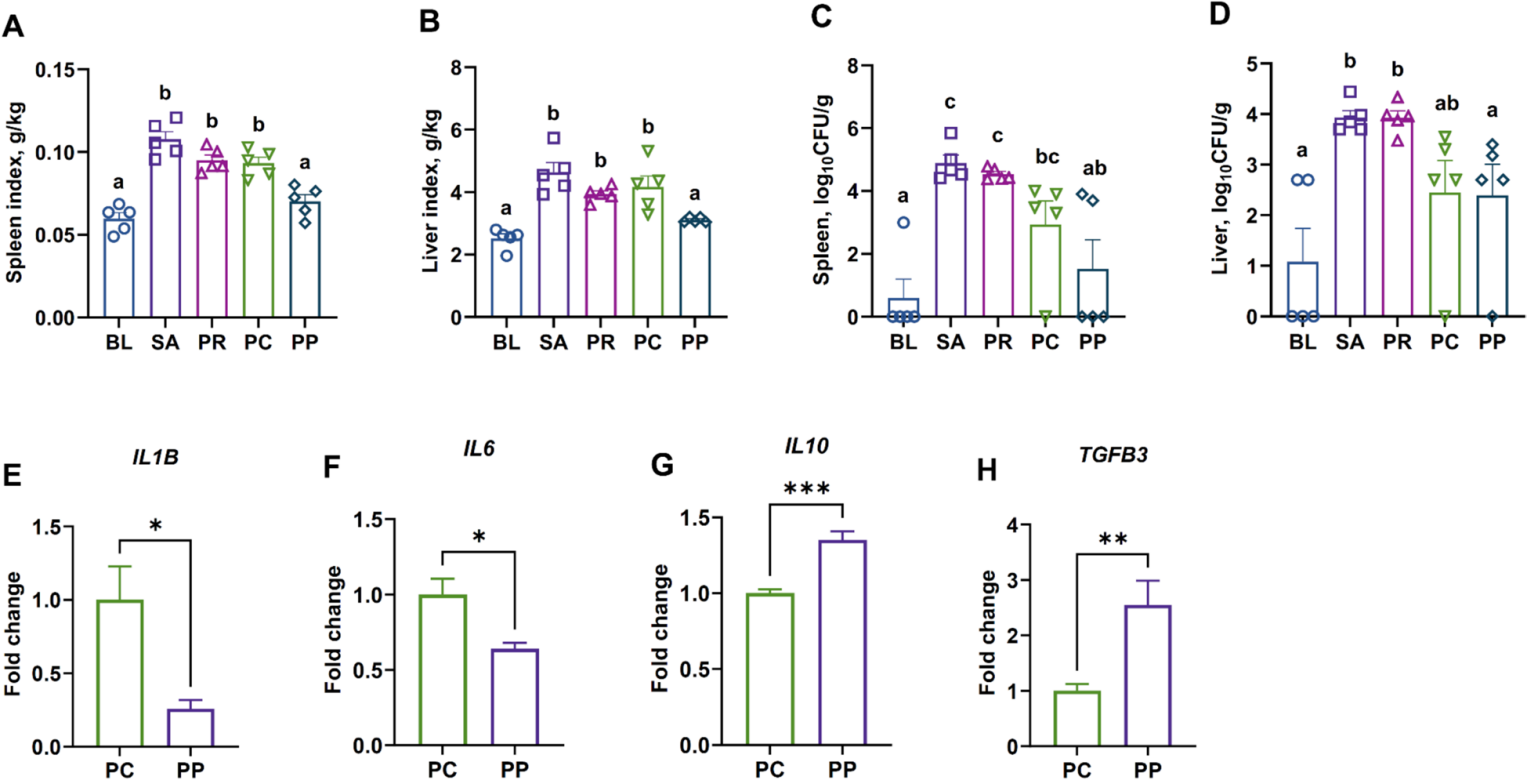
Organ indices, *Salmonella* burden, and splenic cytokines. **A–B** Spleen index and liver index on d 12. Infection (SA) caused splenomegaly and hepatomegaly relative to the uninfected control (BL). The PP treatment prevented this organ enlargement, maintaining spleen and liver sizes comparable to BL, while single treatments (PR, PC) showed higher indices. **C–D** *Salmonella* counts in spleen and liver. The phage-treated groups (PC, PP) showed a reduction in CFU levels by about 1 to 2 logs compared to the SA and PR groups; however, only the PP group exhibited a statistically significant difference. **E–H** Relative mRNA expression of key splenic cytokines on d 12. Phage–probiotic co-treatment (PP) led to an anti-inflammatory cytokine profile: significantly lower pro-inflammatory *IL1B* (**E)** and *IL6* (**F)** levels than phage alone, and higher anti-inflammatory *IL10* (**G)** and *TGFB3* (**H)** levels. Bars represent mean ± SEM (*n* = 5); groups not sharing a lowercase letter differ significantly at *P* < 0.05 (ANOVA with Tukey’s post hoc test). ^*^P < 0.05, ^**^P < 0.01, ^***^P < 0.001

The SA group showed significantly higher spleen (*P* = 0.0002) and liver (*P* < 0.0001) indices than BL. Relative to BL, spleen indices were higher in PR (*P* = 0.0067) and PC (*P* = 0.014). Liver indices were likewise elevated in PR (*P* = 0.0020) and PC (*P* = 0.0015). In contrast, the PP group’s organ indices were not significantly different from those of the BL group (spleen: *P* = 0.89; liver: *P* = 0.80), indicating that the co-treatment of probiotic and phage cocktail effectively prevented the infection-associated organ hypertrophy. Indeed, BL and PP groups had comparably low spleen and liver indices, whereas all other infected groups (SA, PR, and PC) showed significantly elevated values (Fig. 2A and B).

To quantify systemic bacterial dissemination, spleen and liver homogenates were plated on XLD agar to enumerate CFU. In the spleen, *Salmonella* was essentially undetectable in the BL group as expected for uninfected BL group. The PP group similarly had extremely low spleen CFU counts, with no significant difference from the BL group (*P* = 0.64). In contrast, both the SA (*P* < 0.0001) and PR (*P* = 0.0002) groups harbored very high *Salmonella* loads in the spleen—approximately 4-log_10_ CFU greater than the BL baseline (Fig. 2C). Although not explicitly mentioned above, the PC group also achieved low spleen *Salmonella* (*P* = 0.020), similar to those of the PP group (*P* = 0.25), indicating effective control of systemic infection by phage treatment. A comparable pattern was observed in the liver. The BL group showed a very low level of *Salmonella* in the liver (around 1-log_10_ CFU, near the detection limit). The PC (*P* = 0.083) and PP (*P* = 0.10) groups did not differ significantly from BL, both maintaining low liver *Salmonella* levels due to the phage intervention. Conversely, the SA (*P* = 0.0002) and PR (*P* = 0.0002) groups exhibited ~ 3-log_10_ CFU higher *Salmonella* populations in the liver compared to BL (Fig. 2D).

In addition to suppressing organ enlargement, the PP group modulated splenic cytokine expression in a manner consistent with improved immune homeostasis. Compared to the PC group, the PP group showed significantly reduced mRNA levels of pro-inflammatory markers *IL1B* (*P* = 0.013) and *IL6* (*P* = 0.012), while anti-inflammatory cytokines *IL10* (*P* = 0.0004) and *TGFB3* (*P* = 0.0095) were significantly upregulated (Fig. 2E–H). These results indicated that although both PC and PP treatments achieved similarly low levels of systemic bacterial load, the addition of probiotic *L. reuteri* strain LAR11 further mitigated inflammatory responses in the spleen, thereby contributing to the normalization of spleen size and the maintenance of immune balance.

### Phage treatment reduces intestinal *Salmonella* burden regardless of the presence of probiotics

**Fig. 3:**
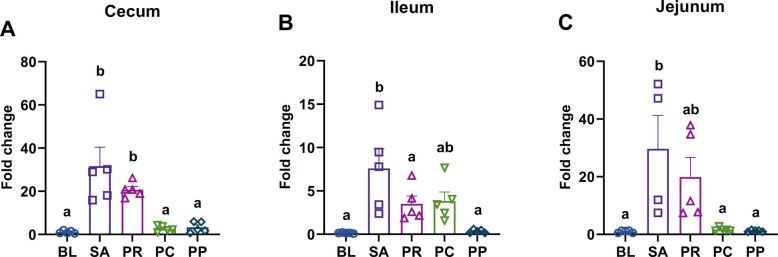
Intestinal *Salmonella* loads in different gut segments. Quantification of *S*. Typhimurium in **A** cecal, **B** ileal, and **C** jejunal contents on d 12 by *invA* gene qPCR. Data are expressed as relative *Salmonella* abundance normalized to total bacterial 16S rRNA gene copies. The uninfected control (BL) and the phage-treated groups (PC and PP) had negligible *Salmonella* in all gut segments. In contrast, the infected control (SA) and the probiotic-only group (PR) showed high *Salmonella* levels (except ileal content). Bars represent mean ± SEM (*n* = 5); groups not sharing a lowercase letter differ significantly at *P* < 0.05 (ANOVA with Tukey’s post hoc test)

*Salmonella* colonization across intestinal segments including cecum, ileum, and jejunum, was quantified by qPCR targeting the *invA* gene, a molecular marker specific to *S.* Typhimurium [50]. In the cecum, *Salmonella* was nearly undetectable in the BL, PC, and PP groups, reflecting either absence of infection (BL) or effective elimination by phages (PC and PP). In contrast, *Salmonella* abundance was significantly elevated in the SA group compared with the BL, PC, and PP groups (*P* = 0.0002, *P* = 0.0003, and *P* = 0.0004, respectively), while the PR group also showed higher levels than BL, PC, and PP (*P* = 0.013, *P* = 0.015, and *P* = 0.019, respectively) (Fig. 3A).

In the ileum, the SA group showed significantly higher *Salmonella* levels than the BL (*P* = 0.0002) and PP (*P* = 0.0003) groups. Unlike the pattern observed in the cecum, the PR group exhibited lower *Salmonella* abundance than the SA group (*P* = 0.042). In contrast, there was no significant difference between SA and PC groups (*P* = 0.067) (Fig. 3B).

In the jejunum, the SA group showed significantly higher *Salmonella* levels than the BL, PC, and PP groups (*P* = 0.038, *P* = 0.045, and *P* = 0.047, respectively), whereas no significant difference was observed between SA and PR (*P* = 0.79) (Fig. 3C). Consistent with the spleen and liver findings, *Salmonella* levels did not differ significantly between the PC and PP groups in any intestinal segment. Both treatments nearly eliminated intestinal *Salmonella*. These results confirm that phage therapy alone was sufficient for intestinal decontamination, with no additional effect from co-treatment with probiotic strain.

### Phage–probiotic co-treatment enhances intestinal morphology and structural integrity compared to phage treatment

**Fig. 4:**
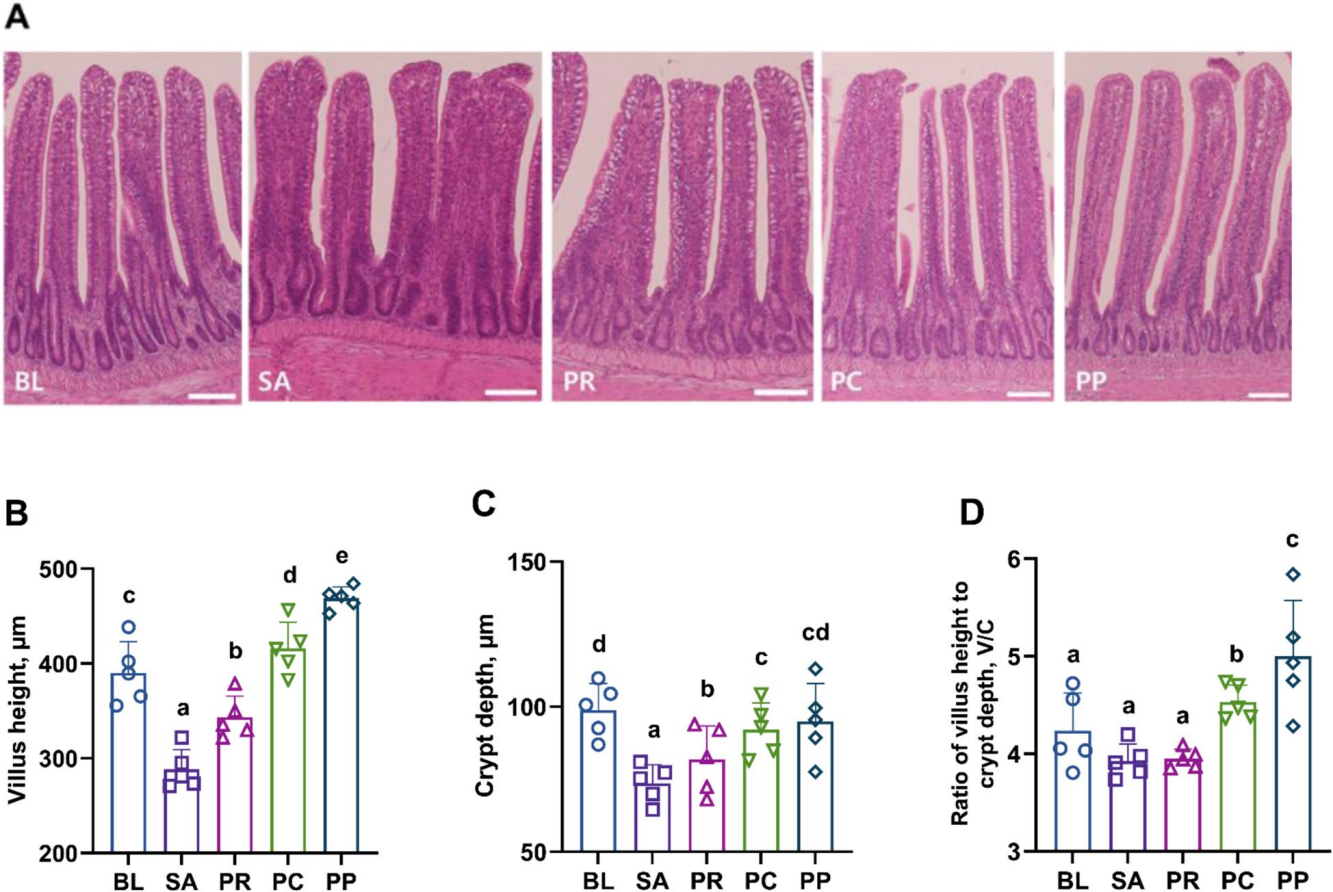
Restoration of distal ileum histology by phage–probiotic treatment. **A** Representative H&E-stained sections of the distal ileum from each group (scale bar = 100 µm). BL (uninfected) shows tall, slender villi and normal crypts. SA (*Salmonella*-infected) exhibits blunted, fused villi and deep, irregular crypts indicative of mucosal damage. PR (probiotic only) and PC (phage only) show partial improvement but still irregular villus architecture. PP (phage + probiotic) displays villi that are elongated and organized, resembling the healthy morphology of BL. **B** Villus height and **C** crypt depth measurements, with **D** the calculated villus height-to-crypt depth ratio for each group. Villus height tended to be highest in the phage-treated groups (PC and PP), while crypt depth was elevated in the BL and PP groups. Notably, the VH/CD ratio was highest in the PP group, whereas the BL, SA, and PR groups showed comparable values. Bars represent mean ± SEM (*n* = 5 birds, averaging ≥ 10 villi per bird); groups not sharing a lowercase letter differ significantly at *P* < 0.05 (ANOVA with Tukey’s post hoc test)

The distal ileum, particularly near the ileocecal junction, represents a pivotal site for nutrient uptake and early *Salmonella* invasion, characterized by abundant Peyer’s patches and M cells [51]. To assess intestinal structural integrity, histological analysis was performed on distal ileal sections, with villus height (VH), crypt depth (CD), and the VH/CD ratio were measured as indicators of mucosal absorptive surface and tissue regeneration capacity. H&E staining revealed stark differences among the groups (Fig. 4A). The BL group had long, slender, and well-organized intestinal villi with uniform crypts, indicative of healthy mucosal architecture. In the SA group, the villi were markedly hypertrophic, blunted, or even fused together, and the crypts appeared irregular and enlarged — pathological changes consistent with inflammation and tissue remodeling due to infection [52]. The PR and PC groups, despite interventions, still showed abnormal villus morphology: their villi were thicker or hypertrophied similar to the SA group, and crypt architecture remained somewhat irregular. Notably, the PP group showed ileal villi and crypt structures that closely resembled those of the healthy BL group. The PP group’s villi were more orderly and elongated, and crypts were more regular, suggesting a near-complete restoration of normal mucosal structure by the co-treatment of probiotic and phage cocktail.

Quantitative measurements of mucosal morphology supported these observations. Villus height was significantly increased in both the PC (*P* = 0.0019) and PP (*P* < 0.0001) groups relative to the BL group (Fig. 4B). This suggests that phage treatment, with or without probiotic, led to elongated villi, potentially as a compensatory response improving absorptive surface area. Crypt depth showed different effects: the PP group’s crypt depth was statistically comparable to that of the BL group (*P* = 0.29), indicating that the crypts remained at normal depth, whereas the PC group (*P* = 0.019) had significantly shallower crypts than BL (Fig. 4C). A reduced crypt depth in the PC group might reflect suppressed crypt hyperplasia or altered tissue turnover after phage treatment alone. The VH/CD ratio as an overall indicator of absorptive efficiency, was highest in the PP group, followed by the PC group (*P* = 0.044) (Fig. 4D). The PP group’s VH/CD was significantly greater than all other groups, while the PC group’s ratio was also elevated compared to the infected controls. The SA, PR, and BL groups showed no significant differences among each other in VH/CD (with SA and PR presumably having low ratios due to villus damage). Collectively, these results indicate that *Salmonella* infection severely impairs intestinal structure, but phage treatment helps to counteract this damage by promoting villus regrowth (and, in the case of phage alone, reducing crypt size). Importantly, the combination of phage and probiotic provided the most effective mucosal protection, preserving crypt depth while enhancing villus height, thereby achieving the most favorable villus-to-crypt ratio.

### Phage–probiotic co-treatment selectively reinforces colonic tight junction integrity compared to phage treatment

**Fig. 5:**
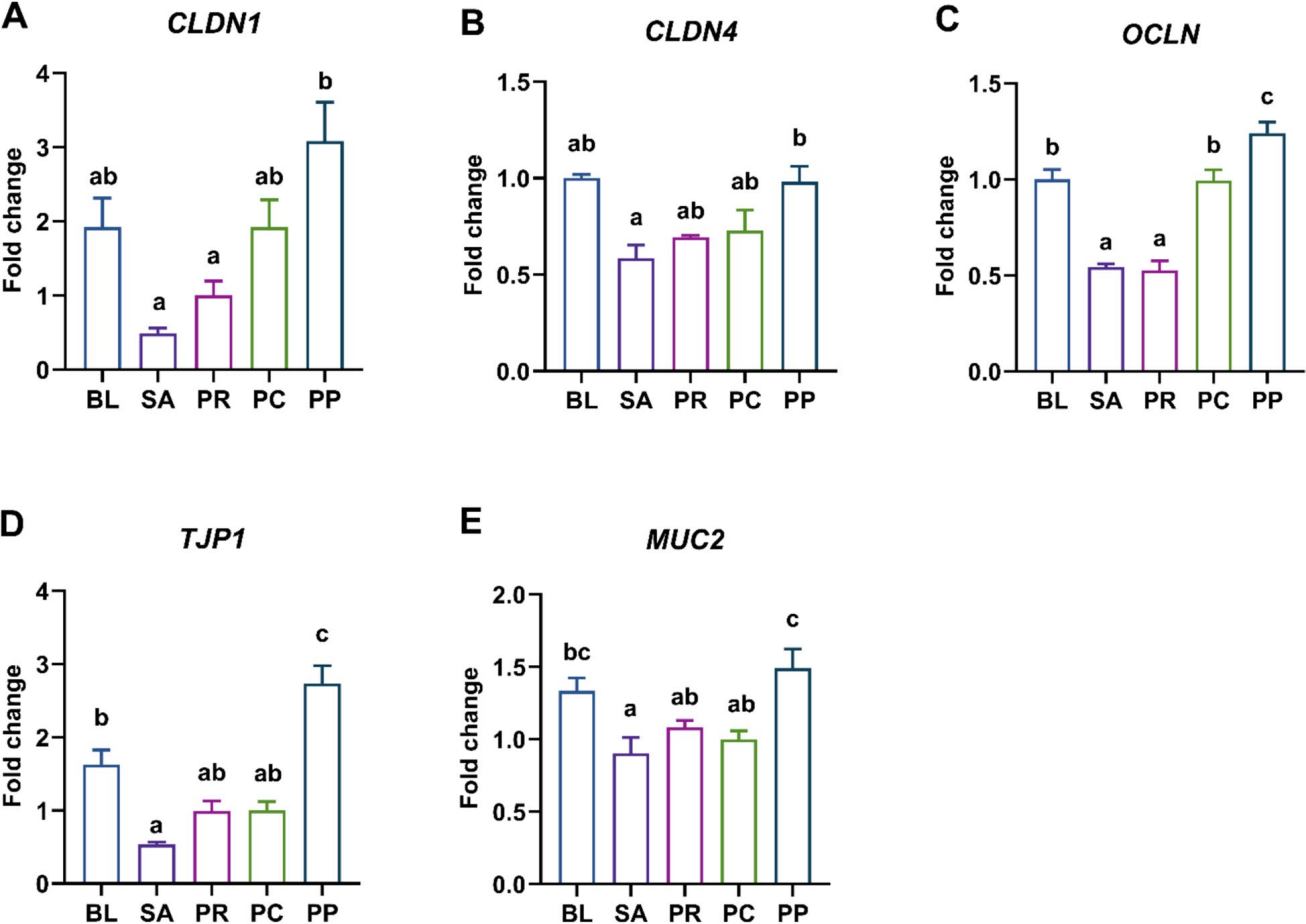
Colonic barrier function gene expression under different treatments. Relative mRNA levels of epithelial tight junction and mucus genes in colonic tissue on d 12. **A** *CLDN1* (Claudin-1), **B** *CLDN4* (Claudin-4), **C** *OCLN* (Occludin), **D** *TJP1* (ZO-1), and **E** *MUC2* (Mucin-2). *Salmonella* infection (SA) downregulated barrier-related genes (*OCLN, TJP1, MUC2*) compared to the uninfected baseline (BL). However, the phage–probiotic combination (PP) significantly upregulated all genes beyond levels seen in SA and in some cases even above BL (*OCLN, TJP1*) and PC (*OCLN, TJP1, MUC2*). Bars represent mean ± SEM (*n* = 5); groups not sharing a lowercase letter differ significantly at *P* < 0.05 (ANOVA with Tukey’s post hoc test)

Expression levels of tight junction proteins and a mucin-related gene in the colon were examined to assess intestinal barrier integrity. The genes quantified included two claudins (*CLDN1* and *CLDN4*), the transmembrane protein occludin (*OCLN*), the scaffold protein ZO-1 (*TJP1*), and the mucus-forming mucin 2 (*MUC2*). For *CLDN1*, the PP group had significantly higher expression levels than both the SA (*P* = 0.0003) and PR (*P* = 0.0034) groups, whereas the BL (*P* = 0.167) and PC (*P* = 0.169) groups did not differ significantly from SA (Fig. 5A). A similar trend was observed for *CLDN4*, where only the PP group demonstrated a significant increase compared to SA (*P* = 0.0045), while the other groups showed similar levels to SA (Fig. 5B). *OCLN* expression was notably diminished in both the SA and PR groups. In contrast, the BL (*P* = 0.016) and PC (*P* = 0.014) groups had significantly higher levels, with the PP group exhibited the highest *OCLN* expression among all treatment groups (Fig. 5C). A comparable pattern was found in the expression of *TJP1*. The PP group showed significantly higher expression than the PC group (*P* < 0.0001), and the BL group also had significantly higher expression than SA (*P* = 0.0011). However, the SA, PR, and PC groups displayed similar expression levels of *TJP1* without significant differences (Fig. 5D). Regarding *MUC2*, the lowest expression levels were recorded in the SA group. Neither the PR (*P* = 0.67) nor PC (*P* = 0.95) groups showed significant differences from SA, whereas both the BL (*P* = 0.029) and PP (*P* = 0.0021) groups had markedly higher expression levels (Fig. 5E).

### Phage–probiotic co-treatment diminishes colonic proinflammatory cytokine expression compared to phage treatment

**Fig. 6:**
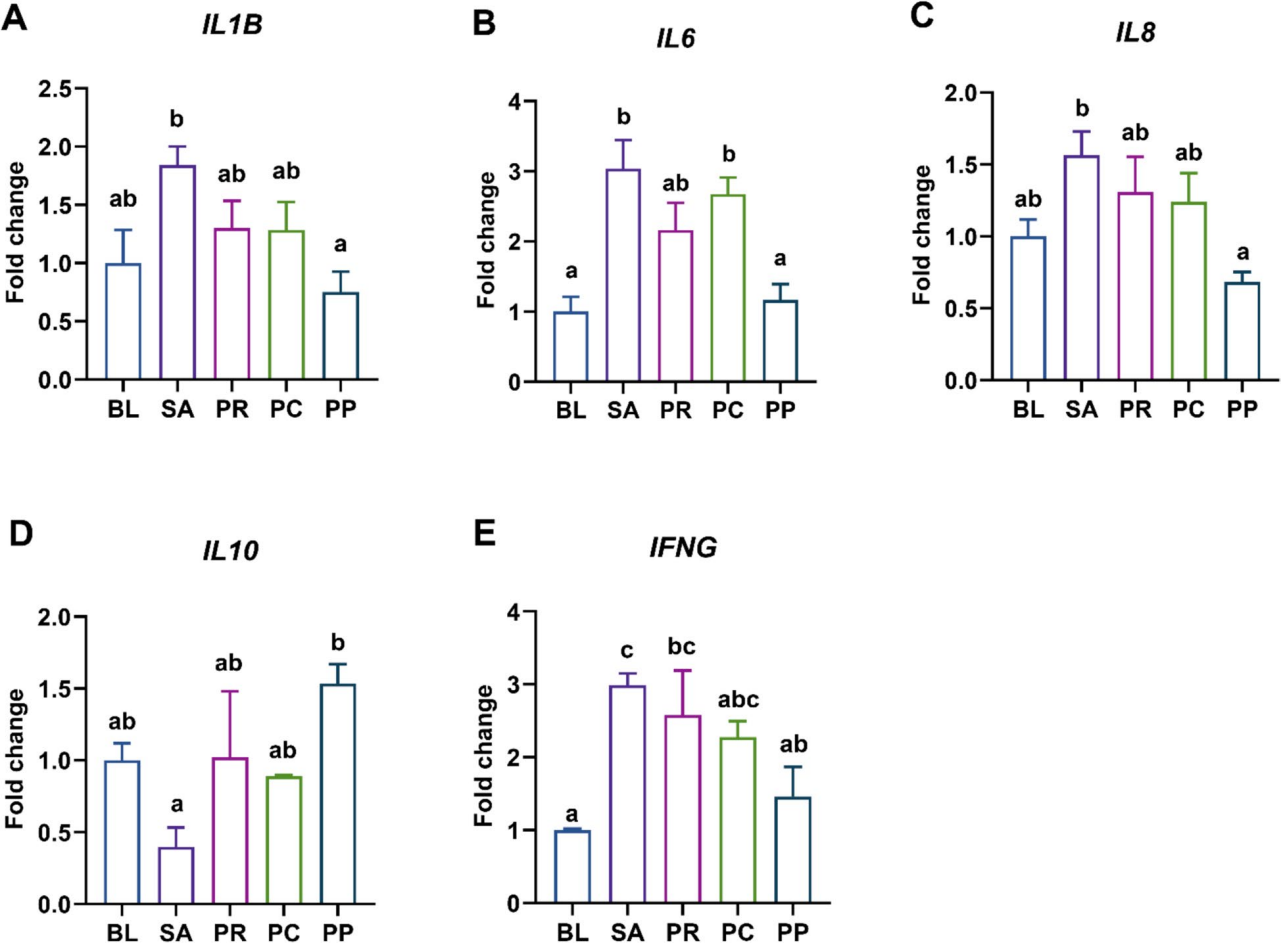
Modulation of colonic inflammatory cytokines by treatments. Relative expression of inflammation-related cytokine genes in the colon on d 12. Pro-inflammatory cytokines: **A** *IL1B* (Interleukin-1β), **B** *IL6* (Interleukin-6), **C** *IL8* (CXCLi2) and **E** *IFNG* (Interferon-γ); and anti-inflammatory cytokine **D** *IL10* (Interleukin-10). *Salmonella* infection (SA) induced colonic inflammation by upregulating *IL6* and *IFNG* expression compared to the uninfected baseline (BL) group. In contrast, all pro-inflammatory cytokines were downregulated in the PP group relative to the SA group, and it was the only group in which anti-inflammatory cytokine expression was significantly upregulated. Bars represent mean ± SEM (*n* = 5); groups not sharing a lowercase letter differ significantly at *P* < 0.05 (ANOVA with Tukey’s post hoc test)

To evaluate inflammatory responses under different treatments, colonic expression levels of key cytokine genes were quantified. The analyzed targets included proinflammatory cytokines *IL1B*, *IL6*, and *IL8* (also referred to as *CXCLi2* in chickens), the anti-inflammatory cytokine *IL10*, and the Th1-associated inflammatory cytokine *IFNG*. Among all groups, the SA group exhibited the highest *IL1B* expression, with only the PP group showing a statistically significant reduction compared to SA (*P* = 0.019). The PR and PC groups did not differ significantly from SA (Fig. 6A). A similar expression pattern was observed for *IL6*, where levels were highest in the SA group. Both the BL (*P* = 0.0011) and PP (*P* = 0.0027) groups exhibited significantly lower *IL6* expression compared to SA, with the PP group also showing a significant reduction relative to the PC group (*P* = 0.018) (Fig. 6B). With respect to *IL8*, the PP group again demonstrated a significant decrease in expression compared to the SA group (*P* = 0.012), while other groups did not (Fig. 6C). Regarding *IL10*, the PP group had the highest expression, but the difference was significant only in comparison to the SA group (*P* = 0.017) (Fig. 6D). In the case of *IFNG*, expression was highest in the SA group, while the BL (*P* = 0.0054) and PP (*P* = 0.041) groups showed significantly reduced levels. No significant differences were observed between SA and either the PR or PC groups (Fig. 6E).

### Phage–probiotic treatment modulates cecal microbial composition by reshaping bacterial abundance

**Fig. 7:**
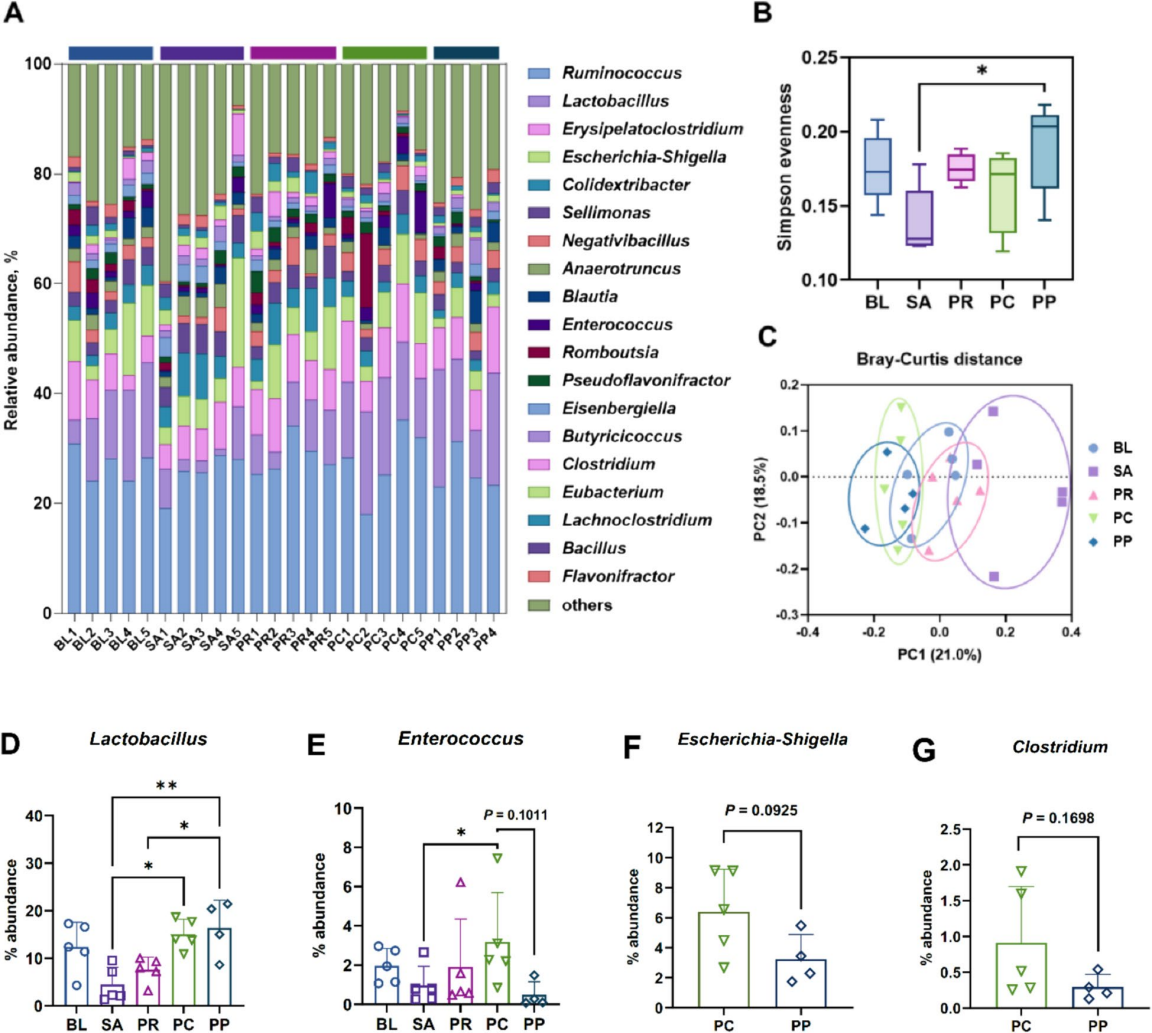
Cecal microbiota composition and diversity across groups. **A** Stacked bar charts showing the genus-level relative abundance of cecal microbiota in each treatment group on d 12. *Salmonella* infection (SA) caused a dysbiosis characterized by a loss of beneficial genera such as *Lactobacillus*. Prophylactic phage (PC) and especially phage + probiotic (PP) maintained a more balanced community, with higher proportions of *Lactobacillus* and other fermentative commensals. **B** Simpson’s evenness index as a measure of alpha-diversity in each group. SA had the lowest evenness, while PP significantly improved microbial evenness toward healthy levels. **C** Principal Coordinates Analysis (PCoA) of Bray–Curtis dissimilarities among cecal samples. The SA group clustered apart from the others, while no clear separation was observed among the treated groups. **D–G** Relative abundances of select bacterial genera critical for gut health or disease. **D** *Lactobacillus—*drastically reduced by *Salmonella* infection (SA) but preserved or enriched in both PC and PP groups (PP showing the highest levels, even above PR). **E** *Enterococcus—*an opportunistic genus which increased notably in the phage-only group (PC) but remained low in PP (similar to SA and BL). **F** *Escherichia-Shigella* and **G** *Clostridium—*pathogenic or opportunistic genera that showed a trend of reduction with combined treatment (PP) compared to PC, though differences were not statistically significant. Statistical analysis was performed using one-way ANOVA with Tukey’s post hoc test. ^*^P < 0.05, ^**^P < 0.01

Cecal microbiota was profiled, as the cecum is the primary fermentation site and the most densely populated microbial niche in the chickens. Taxonomic profiles at the genus level was visualized for each group (Fig. 7A). A marked depletion of *Lactobacillus* was observed in the SA group, whereas both PC and PP groups exhibited significantly higher levels of *Lactobacillus*. Alpha diversity, measured using Simpson’s evenness index, showed the lowest value in the SA group, reflecting severe microbial imbalance. The PP group demonstrated significantly higher evenness compared to SA (*P* = 0.036), indicating improved microbial balance (Fig. 7B). Principal coordinates analysis (PCoA) based on Bray–Curtis dissimilarity did not show distinct clustering with strong statistical separation, but SA samples tended to cluster apart from other groups, while samples from BL, PR, PC, and PP groups were more intermingled (Fig. 7C).

Among key genera, the relative abundance of *Lactobacillus* was significantly lower in the SA group than in both PC (*P* = 0.013) and PP (*P* = 0.0067) groups. Notably, the PP group exhibited a higher *Lactobacillus* level than PR (*P* = 0.048), suggesting that combining phage with probiotic was more effective in supporting this beneficial genus than probiotic alone (Fig. 7D). *Enterococcus*, an opportunistic pathogen, displayed the lowest relative abundance in both SA and PP groups (Fig. 7E). In contrast, the PC group showed a significantly higher abundance of *Enterococcus* compared to SA (*P* = 0.038) and PP (*P* = 0.101), indicating that phage treatment alone may inadvertently allow overgrowth of certain commensals by reducing competition after *Salmonella* elimination. This effect appeared attenuated by probiotic co-treatment. No statistically significant difference was observed between PC and PP groups for pathogenic genera such as *Escherichia–Shigella* and *Clostridium*, although the PP group showed a trend toward lower abundance (Fig. 7F–G). This observation is consistent with previous findings suggesting that combination of phage and probiotic can enhance suppression of non-target pathogenic bacteria, potentially through niche competition and immune modulation [30]. Changes in microbiota composition induced by the phage–probiotic combination extended beyond dominant genera. Among SCFA-producing genera, *Blautia* showed a tendency toward higher abundance in the PP group than in PC (*P* = 0.11), and *Butyricicoccus* was significantly more abundant in PP than in PR (*P* = 0.14) (Fig. S2A–B) [53, 54]. *Eisenbergiella*, a potential probiotic genus known to increase in response to probiotic supplementation, was significantly reduced in the PC group compared to SA (*P* = 0.014), but this reduction was not observed in PP (Fig. S2C) [55]. Conversely, genera associated with intestinal inflammation, such as *Sellimonas* (*P* = 0.022) and *Colidextribacter* (*P* = 0.044), were significantly diminished in the PP group than in SA ( Fig. S2D–E) [56, 57].

### Phage–probiotic co-treatment enhances anti-inflammatory and antimicrobial metabolite production beyond phage treatment

To evaluate whether microbiota and host response changes translated into differences in microbial or host-derived metabolites, a metabolomic analysis was performed on cecal contents. A total of 82 metabolites were quantified and normalized across samples. Heatmap visualization and multivariate analyses were applied to identify group-specific metabolic features.

**Fig. 8:**
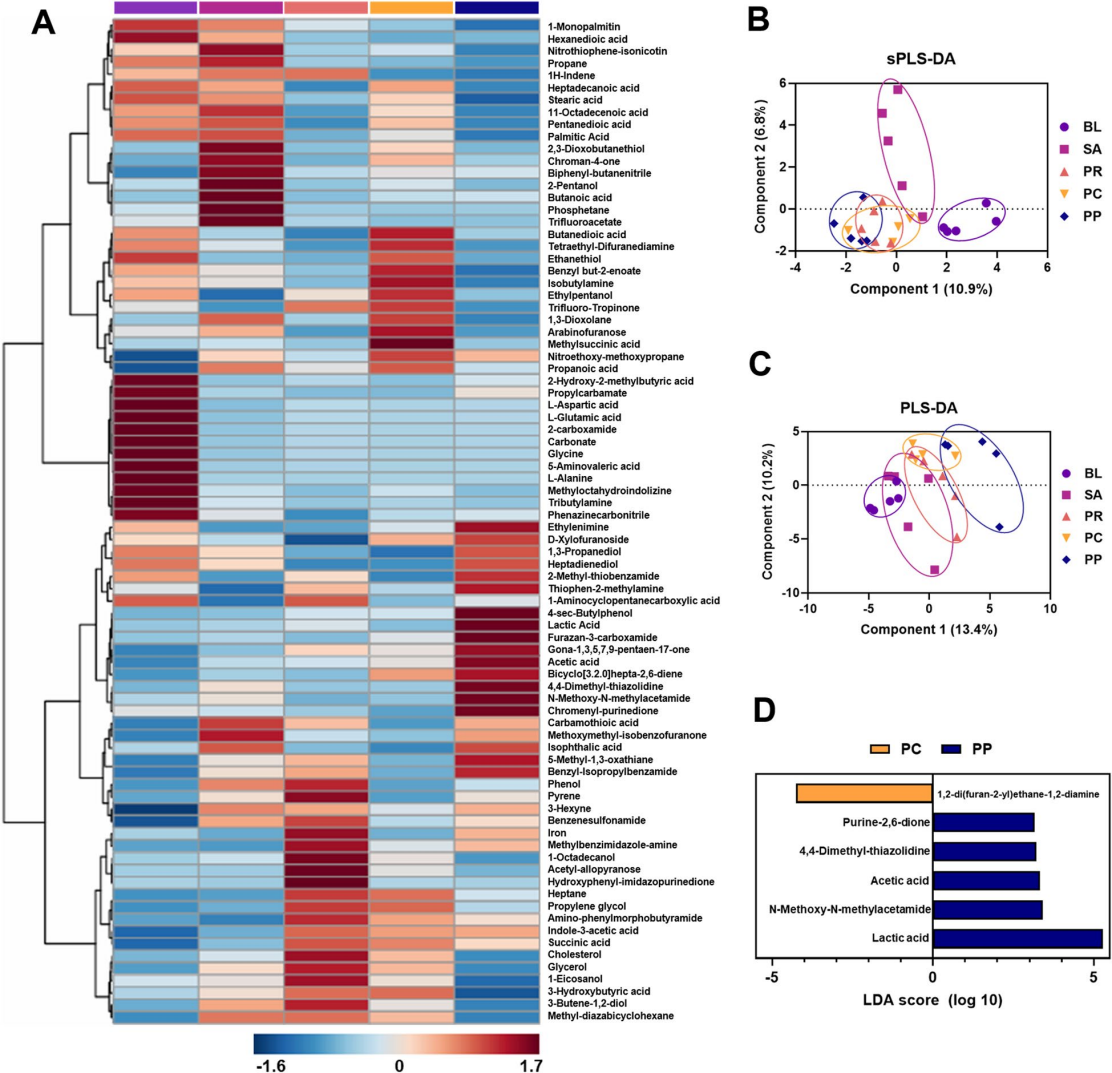
Differential cecal metabolite profiles induced by bacteriophage–probiotic therapy. **A** Heatmap of 82 identified metabolites from cecal content samples (rows) across individual birds (columns) in each group, with hierarchical clustering. Warmer colors indicate higher metabolite levels. The PP group displays a unique cluster of metabolite abundance that differs from both SA and BL. **B** Supervised partial least squares discriminant analysis (sPLS-DA) score plot highlighting group separation in metabolic profiles. BL (uninfected) and SA (infected) form clearly separated clusters. Notably, the PR, PC, and PP groups clustered similarly. **C** Unsupervised PLS-DA score plot showing overall variance in metabolite data. The PP group clustered distinctly from all groups except PC, showing proximity to PC and PR, but clear separation from BL and SA. **D** Linear discriminant analysis effect size (LEfSe) analysis identified several compounds driving the differences between phage-only (PC) and phage + probiotic (PP) treatments. PP group had higher levels of fermentation acids such as acetic acid and lactic acid (key microbial metabolites with antimicrobial and anti-inflammatory properties). Metabolites with an LDA score > 2 are presented

The heatmap revealed distinct clustering patterns across treatment groups (Fig. 8A). Notably, the PP group exhibited a metabolite signature that was most dissimilar to both the BL and SA groups, suggesting a unique metabolic profile induced by the probiotic and phage combination treatment. This observation was further corroborated by supervised partial least squares discriminant analysis (sPLS-DA), which demonstrated clear separation between the BL and SA groups, representing distinct metabolic phenotypes associated with healthy and infected states (Fig. 8B). Among all treatment groups, the PP group was positioned furthest from both BL and SA in sPLS-DA space, indicating a distinct shift in metabolic profile. In contrast, the PR and PC groups showed partial overlap with SA and did not form separate clusters. Unsupervised PLS-DA showed less separation between BL and SA groups, and no statistically significant distinction between SA and either PR or PC. However, the PP group formed a separate cluster, suggesting a distinct metabolic configuration not shared with the other groups (Fig. 8C).

Despite these clustering differences, one-way ANOVA on individual metabolites revealed few statistically significant changes after correction for multiple comparisons. This implies that group differences were driven by broader multivariate shifts rather than large changes in specific metabolites, and that inter-individual variation may have masked some changes. To directly compare the PC and PP groups, linear discriminant analysis (LDA-effect size) was conducted. This identified six metabolites with LDA scores greater than 2. Among them, 1,2-di(furan-2-yl)ethane-1,2-diamine (LDA = 4.27) was enriched in the PC group, while the remaining 5—purine-2,6-dione (LDA = 3.16), 4,4-dimethyl-thiazolidine (LDA = 3.22), acetic acid (LDA = 3.32), N-methoxy-N-methylacetamide (LDA = 3.41), and lactic acid (LDA = 5.29)—were more abundant in the PP group. Acetic acid and lactic acid, both well-characterized fermentation end-products of lactic acid bacteria, are known to exert antimicrobial effects, reduce luminal pH, enhance epithelial barrier integrity, and mediate anti-inflammatory responses [58–61]. The elevated abundance of these compounds in the PP group supports the hypothesis that the combined phage–probiotic treatment promotes a gut environment favorable to fermentation and mucosal health.

### Phage–probiotic co-treatment establishes unique beneficial microbiota–metabolite correlations

**Fig. 9:**
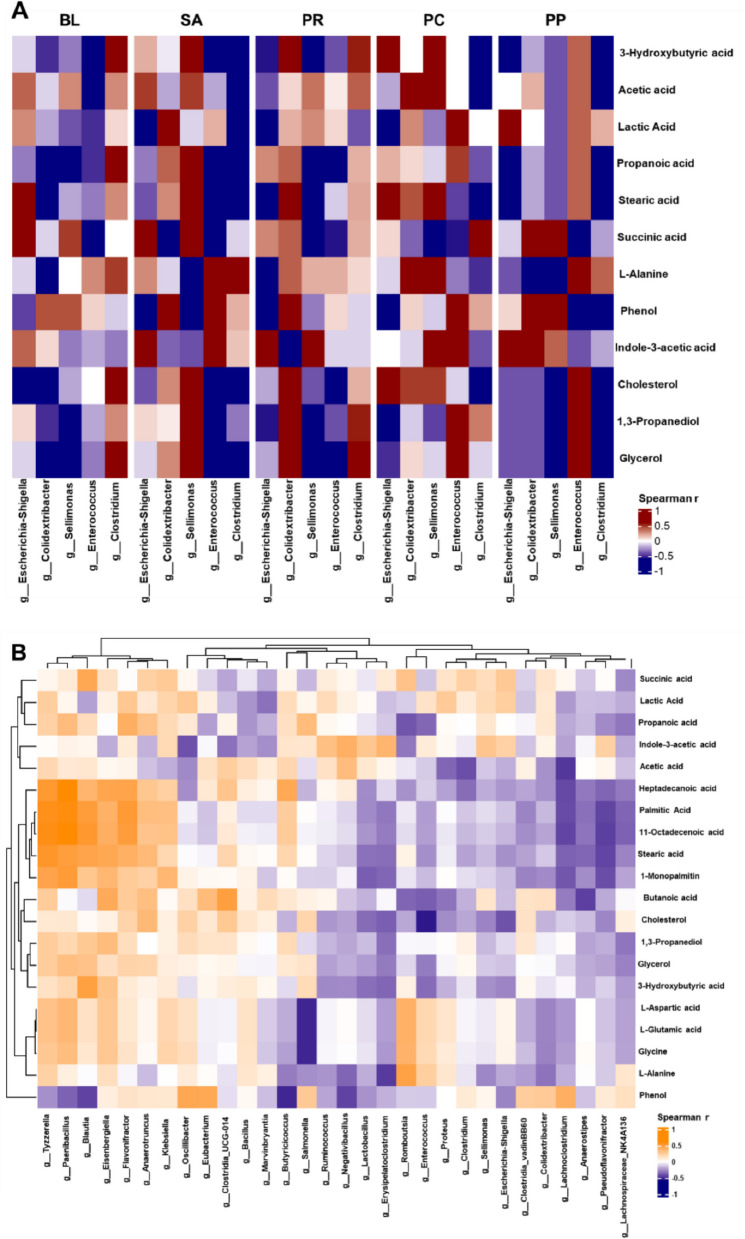
Correlation heatmaps of microbial composition and metabolites in cecal contents **A** Group-specific correlation heatmaps (BL, SA, PR, PC, PP). Spearman correlation matrices between selected microbial composition (e.g., *Lactobacillus, Blautia, Salmonella*, etc.) and key metabolites (including short-chain fatty acids and other significant metabolites) within each experimental group: BL, SA, PR, PC, and PP. The color scale indicates correlation strength (red for positive correlations, blue for negative correlations). This composite figure highlights overall microbiota–metabolite association patterns and how these correlations differ in each treatment group. **B** Global correlation heatmap (all groups combined). Spearman correlation matrix between all detected microbiota and metabolites across all samples, combining all groups

Correlation analysis revealed that chicks receiving the combined PP group developed distinct associations between their gut microbiota and metabolites, which were not observed in other treatments. In the PP group, cecal fermentation acids were strongly linked with beneficial bacterial taxa. For instance, total SCFAs, including acetic and propionic acid, were positively correlated with butyrate-producing commensal genera such as *Butyricicoccus* and *Blautia* (Fig. 9A). These SCFA–microbe relationships were either significantly weaker or entirely lacking in the PC group. SCFAs are vital metabolites for intestinal homeostasis—serving as an energy source for colonocytes and reinforcing gut barrier integrity—so the exclusive SCFA–commensal correlations in PP group indicate an enrichment of beneficial fiber fermentation activity under the dual treatment [62]. Furthermore, only the PP group exhibited a significant positive correlation between the probiotic genus *Lactobacillus* and indole-3-acetic acid (IAA), a tryptophan-derived microbial metabolite. This *Lactobacillus*–IAA association was not evident in phage-only or untreated *Salmonella*-infected controls, suggesting that the presence of both phages and probiotics fostered unique production or preservation of IAA by the gut microbiota. In contrast, the PC group and infected controls showed no such link with IAA, implying diminished levels of this potentially immunomodulatory indole compound in the absence of the combined treatment.

The phage–probiotic strategy also modulated correlations involving protein and lipid metabolites, potentially shifting the intestinal microbiota away from harmful fermentation pathways. Notably, in *Salmonella*-infected control birds, *Salmonella* abundance was positively correlated with phenol levels—a toxic aromatic metabolite of amino acid fermentation. However, in the PP group, this association was abolished or reversed (Fig. 9B). Phenol is a known deleterious end-product of gut microbial tyrosine metabolism that can damage intestinal epithelial cells and impair immune responses [63]. The loss of any *Salmonella*–phenol association in PP birds corresponded with significantly lower *Salmonella* counts and a microbiota less inclined toward proteolytic, phenol-producing activity. Additionally, only PP birds showed unique positive correlations between certain commensal genera (e.g., *Butyricicoccus* and other *Clostridiales*) and amino acids like L-alanine in cecal contents, a pattern not seen in other groups. This suggests that under PP treatment the microbiota was metabolizing available amino acids more efficiently or benignly, leaving fewer substrates for pathogenic fermentation into toxic compounds.

Distinct lipid-related correlations were also observed. In the PP group, *Lactobacillus* abundance was inversely correlated with luminal cholesterol concentration (Spearman *r* < 0, *P* < 0.05), implying that increased probiotic levels were associated with reduced intestinal cholesterol content. This correlation was absent in the PC and PR groups. Furthermore, commensal bacteria in PP-treated birds exhibited stronger positive correlations with glycerol and its microbial fermentation product, 1,3-propanediol, metabolites associated with lipid and glycerol fermentation, whereas these associations were weak or undetectable in other treatment groups.

## Discussion

Diverse pathogen control strategies involving combination therapies are being actively explored. Notably, co-administration of bacteriophages and antibiotics has been shown to exert synergistic bactericidal effects while mitigating the emergence of antimicrobial resistance [64, 65]. A previous study demonstrated that the concurrent application of a *Salmonella*-specific phage cocktail and bacteriocins effectively suppressed *Salmonella* contamination on poultry meat [66]. These findings suggest that combination approaches may offer broader or more sustained efficacy compared to single interventions. Our study demonstrates that the combined treatment of phages and probiotics (PP) is fundamentally more effective than the phage-only treatment (PC) in suppressing *Salmonella* infection and promoting gut health. The observed synergy of using these complementary biological agents is consistent with current trends in poultry research. For instance, Shaufi and colleagues showed that dietary supplemented with both a phage cocktail and probiotics significantly improved broiler growth performance and favorably modulated gut microbiota diversity, whereas phage or probiotics alone had lesser effects [26]. Furthermore, the combined use of phages and probiotics has been proposed as a promising alternative to antibiotic growth promoters and has been reported to produce better outcomes than single-agent treatments [67, 68]. Previous studies have primarily focused on the direct effects of combining phages and probiotics, such as the reduction of target pathogens, improvements in host growth performance, and shifts in gut microbiota composition. In contrast, our study expands upon this concept by demonstrating that chickens in the PP group showed significantly improved expression of immune-related genes (*IL1B*, *IL6*, *IL10*, *TGFB3*) in the spleen compared to the PC group, which contributed to the alleviation of hepatosplenomegaly and maintenance of immune homeostasis [69, 70]. Moreover, the detection of *Salmonella* in all three intestinal regions (cecum, ileum, and jejunum) confirmed the broad efficacy of the phage cocktail (SLAM_phiST45 and SLAM_phiST56) across the digestive tract. Importantly, we found that the combination treatment enhanced weight gain by significantly increasing the villus height-to-crypt depth ratio in the distal ileum. Supporting this, microbiome analysis of ileal contents revealed a more stable and balanced bacterial composition (Fig. S3A–D), along with a significant increase in beneficial genera (Fig. S3E–G).

One of the newly observed key findings in this study was the pronounced divergence in cecal microbiota composition between the PC and PP groups. Although phage therapy is generally considered to preserve gut microbial balance due to its narrow host specificity compared to antibiotics [8], previous study using an artificial cecum system have suggested that phage monotherapy may have unintended consequences [30]. Although *Salmonella* was effectively suppressed in phage-only treatment, the abundance of beneficial genera such as *Clostridium butyricum* decreased, whereas potentially harmful taxa like *Clostridium perfringens* increased compared to phage-probiotic treatment. Our in vivo findings substantiated these concerns. The PC group showed enrichment of pathogenic taxa alongside depletion of beneficial commensals, whereas the PP group exhibited the opposite pattern—reducing non-target pathogenic genera and increasing beneficial, SCFA-producing taxa—indicative of a more balanced gut microbiota. The PP group showed a tendency toward higher Shannon diversity and significantly greater Simpson evenness, along with elevated levels of beneficial genera including *Lactobacillus*, *Ruminococcus*, and *Blautia*. These microbial shifts correlated strongly with histological improvements in the distal ileum and enhanced weight gain. Furthermore, the positive modulation of microbiota in the ileum and cecum was closely linked to downstream immunological outcomes in the colon [29, 71]. In particular, only the PP group showed significant improvements in the expression of tight junction proteins and pro-inflammatory cytokines when compared to the SA group. Several markers (*OCLN*, *TJP1*, *IL6*) also showed significant differences between the PP and PC groups, emphasizing the synergistic benefit of the combined treatment. In contrast, no significant clustering or compositional shifts were observed in the jejunum (Fig. S4A–F). This may be due to early resolution of inflammation or limited exposure to *Salmonella* in this region by d 5 post-infection. As *Salmonella* predominantly colonizes the distal ileum and cecum, the jejunum may experience only transient perturbations in the early stages of infection [72, 73]. Nevertheless, although not statistically significant, trends in alpha diversity and specific microbial abundances in the jejunum followed a similar direction to those seen in the ileum and cecum. Collectively, these findings demonstrate the robust microbial remodeling induced by PP treatment, with the most pronounced effects observed in the cecum and ileum. This highlights the importance of analyzing multiple gut regions in microbial intervention studies, as different segments may respond uniquely to the same treatment [74]. In the cecum, the PP group led to a notable shift in fermentation activity, as evidenced by significantly higher levels of SCFAs and related organic acids compared to the PC group. In particular, acetic acid and lactic acid concentrations were markedly elevated in PP birds relative to PC, indicating that the dual treatment promoted microbial fermentation by beneficial gut bacteria. Intriguingly, acetate levels in the PP group were even significantly higher than those in the BL group (Fig. S5A), whereas neither PC nor PR produced such an increase (Fig. S5B–C). This suggests a unique synergistic effect that arises only when phage and probiotic are administered together. The phage cocktail may reduce the *Salmonella* burden and free up ecological niches, while the probiotic provides fermentative microbes that convert dietary substrates into SCFAs. Indeed, previous work in poultry has found that phage–probiotic co-treatment can enrich cecal bacteria known to produce SCFAs (e.g., *Bacteroides*, *Ruminococcus*, *Faecalibacterium*) which ferment polysaccharides into acids like acetate, propionate, and butyrate [26]. It has been proposed that such combinations supply additional bacterial adhesion sites and nutrients, and reduce pathogen-derived toxins, thereby favoring the colonization of SCFA-producing commensals. In contrast, either intervention alone may be insufficient to induce these beneficial microbes or their metabolites at the same magnitude. The elevated lactic acid in PP is also telling, as it likely reflects the metabolic activity of the administered lactic acid bacteria. This probiotic-derived lactic acid, alongside acetate produced by fermenters, contributes to a more acidic cecal environment. Importantly, the metabolic changes observed in the PP group were closely associated with improved host health indicators [75]. Within the PP-treated birds, individuals exhibiting higher cecal acetate and lactate levels tended to have a lower systemic *Salmonella* load and showed reduced expression of inflammatory cytokine genes in the colon as measured by RT-qPCR. This correlation suggests that SCFAs and related metabolites may be mediating protective effects on the host. There is strong evidence that SCFAs serve as key signaling molecules that bolster mucosal defenses and modulate inflammation. SCFAs such as acetate, propionate, and butyrate are known to strengthen the intestinal barrier by serving as energy sources for epithelial cells and by upregulating tight junction proteins, thereby improving gut barrier function [62]. In our context, acetate elevation in PP could have promoted the expression of tight junction genes, helping to repair or maintain the intestinal lining after *Salmonella*-induced damage. Concurrently, SCFAs exert potent immunoregulatory functions. They bind to G-protein coupled receptors on immune cells (notably GPR43/FFAR2 and GPR41/FFAR3) and activate anti-inflammatory signaling pathways. Acetate, for example, engaging GPR43 has been shown to suppress colonic inflammation in experimental models, activation of the acetate–GPR43 axis reduced proinflammatory cytokine production and inflammasome activation in the colon [76, 77]. These mechanisms could explain the lower inflammatory cytokine levels we observed in the PP group’s colonic tissue. By enhancing mucosal barrier integrity and dialing down excessive inflammation, SCFAs create conditions that favor healing and homeostasis. Taken together, the ability of the PP treatment to raise beneficial metabolites likely contributed to faster recovery from infection by tightening the gut barrier against the translocation of bacteria and endotoxins and by modulating the immune response toward the resolution of inflammation [78]. This is consistent with the broader understanding that SCFA–GPCR interactions maintain colonic integrity by promoting mucosal healing and limiting inflammatory damage. Thus, the improved clinical outcomes and health markers in the PP group can be plausibly linked to the advantageous metabolic environment established in the gut.

Beyond the observed increases in SCFA levels, the phage–probiotic combination also impacted tryptophan metabolism in ways that likely contributed to its protective effects. Notably, PP-treated chicks harbored probiotic-associated microbes that were positively correlated with indole-3-acetic acid (IAA), an indole metabolite uniquely elevated in this group. Such a correlation suggests that *Lactobacillus* or other commensals introduced or enriched by the probiotic were actively producing IAA in the presence of phage treatment. IAA and indole derivates are well-recognized microbial byproducts that can modulate mucosal immunity by activating aryl hydrocarbon receptors on host cells [63]. Consistent with this, exogenous IAA administration in animal models has been shown to suppress pro-inflammatory cytokines (like *IL1B*, *IL6* and *IL8*) while upregulating the anti-inflammatory cytokine *IL10* and strengthening the intestinal barrier [79]. Therefore, the elevated IAA levels associated with *Lactobacillus* in PP treated birds likely contributed to an anti-inflammatory intestinal environment and supported mucosal repair. In contrast, *Salmonella*-challenged birds lacking the combined treatment had lower IAA and no beneficial microbe–IAA correlations, indicating the absence of this indole-mediated immunomodulatory pathway. The PP group’s ability to promote indole-producing microbes thus provided an additional layer of immunomodulation that phage therapy alone did not achieve. By enhancing both SCFA and indole production, the phage–probiotic strategy supplied complementary metabolites that reinforce the intestinal barrier and attenuate inflammatory responses, accelerating recovery from *Salmonella*-induced gut injury [63, 79].

The combined treatment also appeared to divert the cecal metabolic profile away from pathogenic proteolysis and toward more beneficial routes. In untreated or singly treated birds, protein fermentation by dysbiotic bacteria can yield aromatic toxins (e.g., phenol, p-cresol) that injure the gut lining and provoke inflammation [63]. Phenolic metabolites are broadly recognized as cytotoxic and pro-inflammatory within the gastrointestinal tract [63]. Our correlation data indicate that the PP intervention mitigated such harmful processes: unlike infected controls, PP birds showed no positive correlation between *Salmonella* and phenol, reflecting a reduction in both *Salmonella* burden and the associated production of phenolic byproducts. This likely resulted from phages directly lysing *Salmonella* (removing a major source of proteolytic activity) in tandem with probiotics outcompeting or suppressing other phenol-producing microbes. These effects would be less accumulation of toxic metabolites that can weaken the intestinal barrier and exacerbate inflammation. Moreover, the unique correlations observed in PP birds with metabolites like L-alanine suggest a redirection of amino acid metabolism toward microbial uptake and utilization of amino acids for growth rather than proteolytic putrefaction. Another intriguing benefit of the phage–probiotic co-treatment was its influence on lipid metabolism in the gut. Only the PP-treated group exhibited a significant inverse correlation between *Lactobacillus* abundance and cecal cholesterol levels, implying that administered lactobacilli may have actively assimilated or co-metabolized cholesterol within the intestinal lumen. Certain *Lactobacillus* strains are known to absorb dietary cholesterol and facilitate its co-precipitation with bile, effectively reducing cholesterol availability [80]. In the PP group, such probiotic activity could have modulated the bile acid pool and created a less hospitable environment for bile-tolerant pathogens, adding another indirect mechanism by which gut pathogens were kept in check. Additionally, the enhanced microbial conversion of glycerol to 1,3-propanediol observed only in PP birds points to a fermentative ecosystem restored to a healthy function when *Salmonella* is held at bay. All of these metabolic shifts including increased SCFA and indole production, reduced phenolic toxin generation, and normalized lipid fermentation, would contribute to a more resilient, less inflammatory intestinal environment.

In summary, the combined phage–probiotic regimen concurrently mitigated *Salmonella* infection across the spleen, liver, cecum, ileum, and jejunum while enriching commensal taxa and metabolites that reinforce epithelial barrier function and attenuate inflammation, thereby improving growth performance. Extending beyond cecal microbiome assessments, we conducted a comprehensive, multi-site comparison of microbiome profiles in the cecum, ileum, and jejunum under phage monotherapy versus phage–probiotic combination [81–83]. Phage alone elicited unintended reductions in beneficial taxa and enrichment of opportunistic bacteria in the cecum and ileum, whereas co-treatment prevented these shifts and restored microbial homeostasis. To our knowledge, this constitutes the first in vivo evidence in poultry that probiotic supplementation offsets collateral microbial perturbations associated with phage monotherapy, consistent with reports that phage therapy can reshape non-target microbial networks and the metabolomic landscape [84, 85]. Although conducted under controlled conditions, the use of a field-isolated *S*. Typhimurium strain and a broad-host-range phage cocktail preserves practical relevance and supports the phage–probiotic combination as a promising and sustainable biocontrol strategy approach for mitigating *Salmonella* burden in poultry production systems.

## Conclusion

This study demonstrates that combining bacteriophages with probiotics exerts synergistic benefits for controlling multidrug-resistant *Salmonella* infections in poultry. While phage treatment alone effectively suppresses pathogen burden, it also induced collateral alterations in the gut microbiota, including the expansion of opportunistic taxa and depletion of beneficial commensals. In contrast, the phage–probiotic combination not only eliminated *Salmonella* but also restored microbial balance, improved intestinal morphology, and promoted immune homeostasis. These effects were associated with enhanced production of short-chain fatty acid and improved growth performance. Importantly, the combination treatment enriched SCFA-producing commensals and probiotic *Lactobacillus* that contribute beneficial metabolites such as butyrate and indole-3-acetic acid, both of which are known to reinforce the intestinal barrier and attenuate inflammatory signaling. Taken together, our findings support phage–probiotic co-treatment as a promising antibiotic-alternative strategy for controlling enteric infections while preserving gut health in poultry. Further validation in commercial settings will be essential to assess its potential for sustainable animal production.

## Supplementary Information

Additional file 1: Table S1. Antibiotic resistance geneprofile of *S*. Typhimurium ST422.

Additional file 2: Fig. S1. Genomic characterization of bacterial strains used. Fig. S2. Effects of treatments on specific cecal bacterial genera. Fig. S3. Ileal microbiota differences between phage-only and phage+probiotic groups. Fig. S4. Jejunal microbiota analysis for phage vs. phage+probiotic treatments. Fig. S5. Cecal short-chain fatty acid levels under different treatments.

## Data Availability

WGS data have been deposited in NCBI GenBank (phage accession numbers PP948674.1 and PP948675.1; the accession number for LAR11 is CP196337). The sequencing data generated in this study have been deposited in the NCBI Sequence Read Archive (SRA) under BioProject accession PRJNA1291055. This includes 16S rRNA gene sequencing reads from cecal content samples of all 5 groups (BL, SA, PR, PC, PP) and from ileal and jejunal content samples of the PC and PP groups. These datasets are publicly available from NCBI. All data needed to evaluate the conclusions in the paper are present in the manuscript. Additional data are available from the authors upon request.

## Abbreviations

ADFI: Average daily feed intake
ARGs: Antibiotic resistance genes
*CLDN1*: Claudin-1 (tight junction protein gene)
*CLDN4*: Claudin-4 (tight junction protein gene)
FCR: Feed conversion ratio
*GAPDH*: Glyceraldehyde-3-phosphate dehydrogenase (housekeeping gene)
GPR41 (FFAR3): G protein-coupled receptor 41 (free fatty acid receptor 3)
GPR43 (FFAR2): G protein-coupled receptor 43 (free fatty acid receptor 2)
H&E: Hematoxylin and eosin (stain)
IAA: Indole-3-acetic acid
*IFNG*: Interferon-gamma (Th1 cytokine gene)
*IL1B*: Interleukin-1 beta (pro-inflammatory cytokine gene)
*IL6*: Interleukin-6 (pro-inflammatory cytokine gene)
*IL8*: Interleukin-8 (CXCLi2, pro-inflammatory cytokine gene)
*IL10*: Interleukin-10 (anti-inflammatory cytokine gene)
MDR: Multidrug-resistant
*MUC2*: Mucin 2 (intestinal mucus glycoprotein gene)
*OCLN*: Occludin (tight junction protein gene)
SCFA: Short-chain fatty acid
*TGFB3*: Transforming growth factor beta 3 (anti-inflammatory cytokine gene)
*TJP1*: Tight junction protein 1 (ZO-1 gene)
WGS: Whole-genome sequencing
