# Supplementary material for: Combination of bacteriophage–probiotics alleviates intestinal barrier dysfunction by regulating gut microbiome in a chick model of multidrug-resistant Salmonella infection
Source: J Anim Sci Biotechnol. 2026 Jan 23;17:14. doi: 10.1186/s40104-025-01324-4 (PMC12829087; doi:10.1186/s40104-025-01324-4)
Supplement: Supplementary file 1 — Additional file 1: Table S1. Antibiotic resistance geneprofile of S. Typhimurium ST422. [file 40104_2025_1324_MOESM1_ESM.docx]

Additional file 1

**Combination of bacteriophage–probiotic alleviates intestinal barrier dysfunction by regulating gut microbiome in a chick model of multidrug-resistant *Salmonella* infection**

Youbin Choi^1^, Anna Kang^1^, Eunsol Seo^1^, Daniel Junpyo Lee^1^, Junha Park^1^, Yeonsoo Kim^1^, Keesun Yu^1^, Cheol‑Heui Yun^1^, Ki Beom Jang^1^, Woo Kyun Kim^2^, Kwanseob Shim^3^ and Darae Kang^3*^, and Younghoon Kim^1*^

^1^Department of Agricultural Biotechnology and Research Institute of Agriculture and Life Science, Seoul National University, Seoul 08826, Korea

^2^Department of Poultry Science, University of Georgia, Athens, GA 30602, United States

^3^Department of Animal Biotechnology, Jeonbuk National University, Jeonju 54896, Korea

*To whom correspondence should be addressed: drkang@jbnu.ac.kr and ykeys2584@snu.ac.kr

**Table S1. Antibiotic resistance gene (ARG) profile of *S*. Typhimurium ST422.** The multidrug-resistant (MDR) ST422 strain harbored 45 antibiotic resistance genes (ARGs) spanning eight antibiotic classes, as determined by Comprehensive Antibiotic Resistance Database (CARD) analysis. The table lists each antibiotic class, the number of ARGs detected, and representative resistance genes, including determinants for fosfomycin, macrolides, tetracyclines, aminoglycosides, β-lactams, polymyxins, glycopeptides, and bacitracin, as well as multiple multidrug efflux pump genes.

| **Antibiotic class** | **Number** | **Genes** |
| --- | --- | --- |
| Fosfomycin | 2 | *UhpT, GlpT* |
| Macrolides | 2 | *Mrx, mphA* |
| Tetracyclines | 1 | *tet(A)* |
| Aminoglycosides | 4 | *APH(3')-Ia, APH(6)-Id, acrD, AAC(6’)-Iy* |
| Beta-lactams | 1 | *Haemophilus influenzae PBP3* |
| Polymyxins | 2 | *ArnT, ugd* |
| Glycopeptides | 1 | *vanG* |
| Bacitracin | 1 | *bacA* |
| Multidrug (Efflux/Regulator) | 15 | *acrA, acrB, acrD, emrA, emrB, emrR, mdfA, mdtM, mdsA, mdsB, mdsC, KpnE, KpnF, marA, soxR, soxS, marR, acrAB, MdtK* |
| Unknown | 16 | *CRP, EF-Tu, rsmA, PmrF, baeR, mdtB, mdtC, sdiA, msbA, kdpE, leuO, cpxA, golS, emrR, emrB* |
