# Supplementary material for: Combination of bacteriophage–probiotics alleviates intestinal barrier dysfunction by regulating gut microbiome in a chick model of multidrug-resistant Salmonella infection
Source: J Anim Sci Biotechnol. 2026 Jan 23;17:14. doi: 10.1186/s40104-025-01324-4 (PMC12829087; doi:10.1186/s40104-025-01324-4)
Supplement: Supplementary file 2 — Additional file 2: Fig. S1. Genomic characterization of bacterial strains used. Fig. S2. Effects of treatments on specific cecal bacterial genera. Fig. S3. Ileal microbiota differences between phage-only and phage+probiotic groups. Fig. S4. Jejunal microbiota analysis for phage vs. phage+probiotic treatments. Fig. S5. Cecal short-chain fatty acid levels under different treatments. [file 40104_2025_1324_MOESM2_ESM.docx]

Additional file 2

**Combination of bacteriophage–probiotic alleviates intestinal barrier dysfunction by regulating gut microbiome in a chick model of multidrug-resistant *Salmonella* infection**

Youbin Choi^1^, Anna Kang^1^, Eunsol Seo^1^, Daniel Junpyo Lee^1^, Junha Park^1^, Yeonsoo Kim^1^, Keesun Yu^1^, Cheol‑Heui Yun^1^, Kibeom Jang^1^, Woo Kyun Kim^2^, Kwanseob Shim^3^ and Darae Kang^3*^, and Younghoon Kim^1*^

^1^Department of Agricultural Biotechnology and Research Institute of Agriculture and Life Science, Seoul National University, Seoul 08826, Korea

^2^Department of Poultry Science, University of Georgia, Athens, GA 30602, United States

^3^Department of Animal Biotechnology, Jeonbuk National University, Jeonju 54896, Korea

*To whom correspondence should be addressed: drkang@jbnu.ac.kr and ykeys2584@snu.ac.kr


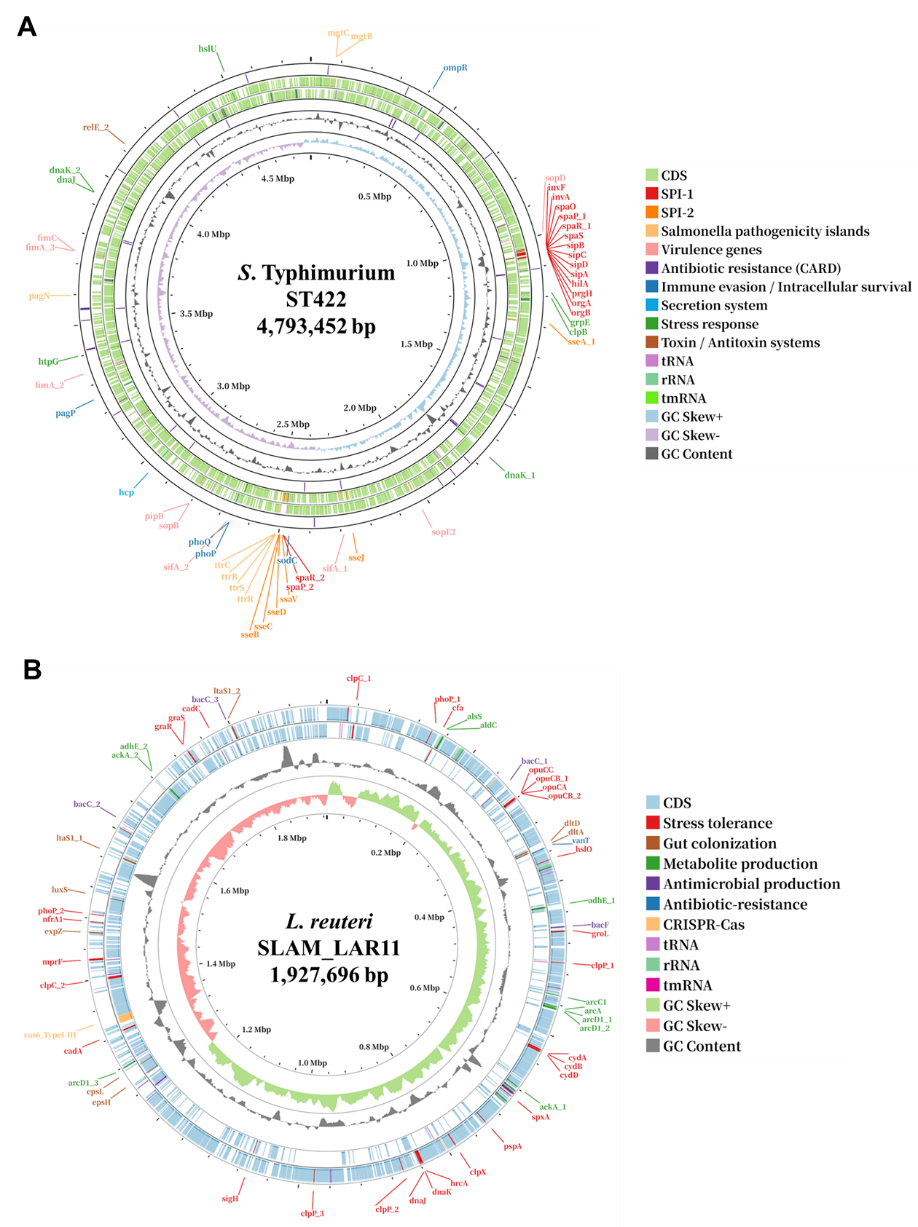


**Fig. S1. Genomic characterization of bacterial strains used. (A)** Circular genome map of *S*. Typhimurium ST422**.** Antibiotic resistance gene profile of *S*. Typhimurium ST422 from whole-genome sequencing, analyzed via the CARD database. Key phenotypic features characteristic of *Salmonella* were also identified. **(B)** Circular genome map of *L. reuteri* LAR11. Key genomic features of the probiotic *L. reuteri* LAR11. Antibiotic resistance gene profile of *L. reuteri* LAR11 from whole-genome sequencing, analyzed via the CARD database. Notably, LAR11 carries the *vanT* gene, which confers intrinsic resistance to vancomycin in lactobacilli. This intrinsic vancomycin resistance is non-transmissible and is considered safe (not a public health concern).

**Fig. S2. Effects of treatments on specific cecal bacterial genera.** Relative abundance of selected genera in the cecal microbiota on day 12. **(A)** *Blautia* – a beneficial SCFA-producing genus showing a higher mean proportion in PP vs. PC (trend, not significant). **(B)** *Butyricicoccus* – a butyrate-producing genus increased in PP compared to the probiotic-only group (PR). **(C)** *Eisenbergiella* – a commensal genus that was significantly reduced by phage-only treatment (lower in PC vs SA), whereas the PP group preserved its abundance. **(D)** *Sellimonas* – an inflammation-associated genus that bloomed under *Salmonella* infection; its levels were significantly reduced in PP birds relative to SA, indicating effective suppression. **(E)** *Colidextribacter* – another genus linked to intestinal inflammation or dysbiosis, also significantly decreased in PP vs SA. Statistical analysis was performed using one-way ANOVA with Tukey’s post hoc test. **P* < 0.05.

**
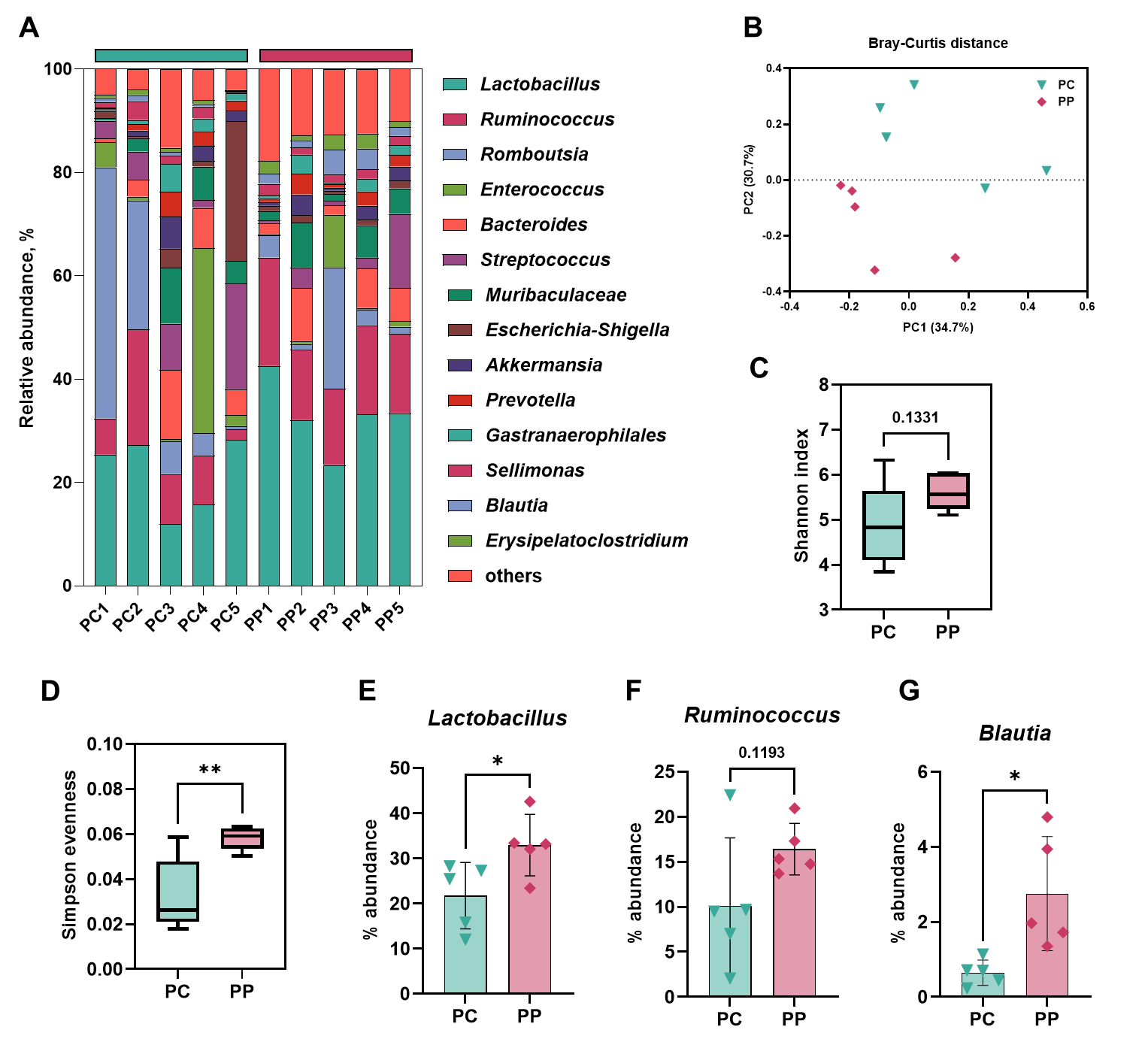
**

**Fig. S3. Ileal microbiota differences between phage-only and phage+probiotic groups.** 16S rRNA sequencing analysis of ileal content samples (day 12) for the PC and PP groups.

**(A)** Stacked bar charts showing the genus-level relative abundance of ileal microbiota in PC and PP groups on day 12. Compared with the cecal microbiota, individual birds showed greater variability in ileal bacterial composition. In the phage-only group, PC1 and PC2 were dominated by *Romboutsia*, PC4 by *Enterococcus*, and PC5 by *Escherichia–Shigella*, indicating an unintended increase in opportunistic bacteria following phage monotherapy. **(B)** Principal coordinates analysis of β-diversity (Bray–Curtis) revealed distinct clustering between the ileal communities of PC and PP, in contrast to the cecal content. **(C-D)** Alpha-diversity metrics in the ileum: the PP group tended to have higher diversity than PC (Shannon diversity index in B showed an upward trend; Simpson’s evenness in C was significantly greater in PP), suggesting a more balanced ileal microbiome with the combined treatment. **(E–G)** Proportions of key beneficial genera in the ileum: *Lactobacillus* (E), *Ruminococcus* (F), and *Blautia* (G). All three showed higher mean abundance in PP-treated birds compared to phage alone, with *Lactobacillus* and *Blautia* notably enriched under the combined treatment. These findings align with cecal data, indicating that PP therapy better supports beneficial microbes even in the upper intestinal regions. Statistical analysis was performed using one-way ANOVA with Tukey’s post hoc test. **P* < 0.05.

**
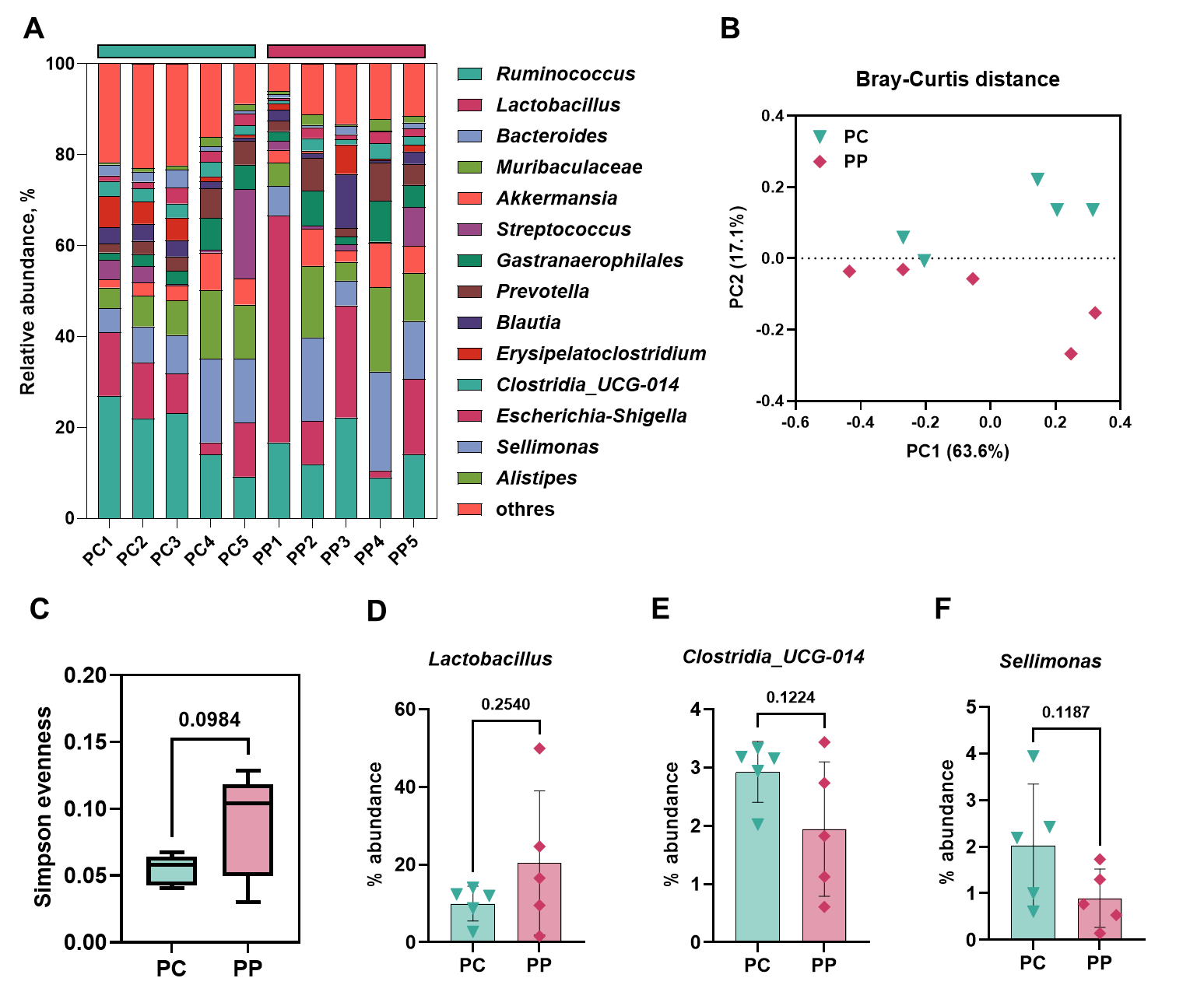
**

**Fig. S4. Jejunal microbiota analysis for phage vs. phage+probiotic treatments.** 16S rRNA sequencing analysis of ileal content samples (day 12) for the PC and PP groups. **(A)** Stacked bar charts showing the genus-level relative abundance of jejunal microbiota in PC and PP groups on day 12. Unlike the cecal and ileal microbiota, no clear consistency was observed among groups. **(B)** Principal coordinates analysis of β-diversity (Bray–Curtis) showed no distinct clustering. (**C)** Simpson’s evenness in the jejunal microbiome showed a similar pattern to the ileum and cecum – the PP group had slightly higher diversity and evenness than PC, though differences were not statistically significant due to greater variability and lower overall bacterial load in this section. **(D–F)** Relative abundances of representative genera in the jejunum mirrored trends seen in other gut sections: *Lactobacillus* (D) was somewhat higher in PP than PC; minor opportunists such as Enterococcus or *Escherichia* (data not shown explicitly) remained low in both groups; and SCFA-producers like *Ruminococcus* or *Blautia* (E, F) showed slight increases with PP.


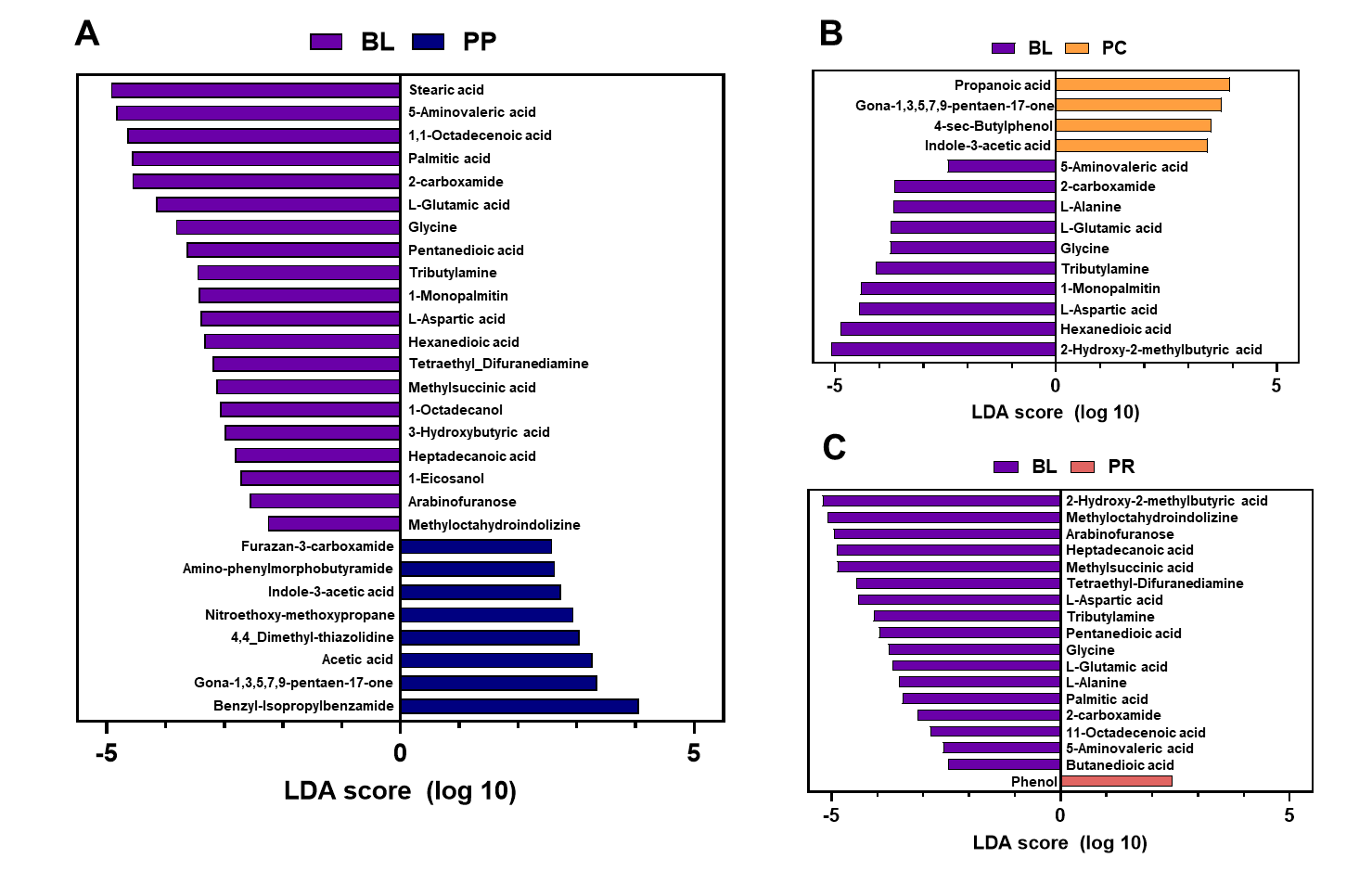


**Fig. S5. Cecal short-chain fatty acid levels under different treatments.** Concentrations of key SCFAs in cecal contents on day 12, comparing treatment effects. **(A)** Linear discriminant analysis effect size (LEfSe) analysis identified several compounds driving the differences between uninfected baseline (BL) and phage+probiotic (PP) treatments. **(B)** LEfSe analysis identified several compounds driving the differences between BL and phage-only (PC) treatments. **(C)** LEfSe analysis identified several compounds driving the differences between BL and probiotic-only (PR) treatments. Acetic acid levels were significantly higher in the PP group compared with both BL and PC, whereas no such increase was observed when comparing BL with PC or PR, indicating that the elevation of this metabolite is specific to PP treatment. Metabolites with an LDA score > 2 are presented.
